# Multifunctional Nanovaccine‐Radiotherapy Synergy Drives Anti‐Tumor Immunity via Multi‐Cascade Cell Death for Pancreatic Cancer Therapy

**DOI:** 10.1002/advs.77651

**Published:** 2026-09-10

**Authors:** Chengyi Huang, Yushu Han, Xiang Liu, Chuanhao Wang, Xiaofei Zhu, Di Chen, Yangyang Gong, Liang Chen, Wenjuan Chen, Zhenyuan Miao, Bufu Tang, Huojun Zhang

**Affiliations:** ^1^ Department of Radiation Oncology Changhai Hospital affiliated to Naval Medical University Shanghai China; ^2^ Department of Oncology Zhujiang Hospital Southern Medical University Guangzhou Guangdong China; ^3^ School of Pharmacy Naval Medical University Shanghai China; ^4^ Department of Interventional Radiology Shanghai Institute of Medical Imaging Shanghai Institution of Medical Imaging Zhongshan Hospital, Shanghai National Clinical Research Center of Interventional Medicine Fudan University Shanghai China

**Keywords:** ferroptosis, FSTL3, immunogenic cell death, nanovaccine, pancreatic cancer, radiotherapy, tumor microenvironment

## Abstract

Pancreatic adenocarcinoma (PAAD) is an immunosuppressive malignancy refractory to radiotherapy and immunotherapy. Conventional tumor vaccines fail in PAAD due to a suppressive tumor microenvironment (TME), inadequate antigen presentation, and impaired immunogenic cell death (ICD). Using a machine learning‐driven transcriptomic analysis, we identified FSTL3 as a vital TME regulatory factor enriched in desmoplastic pancreatic lesions and constructed a cRGD‐modified liposomal carrier co‐encapsulating gemcitabine and siFSTL3 (siFSTL3/Lip_GEM_‐cRGD). Unlike antigen‐preloaded conventional nanovaccines, this liposome acts as an irradiation‐activated inducer of in situ tumor vaccination. This engineered multifunctional nanovaccine enhanced radiochemosensitivity and, combined with ionizing radiation (IR), promoted dendritic cell maturation, ICD, and ferroptosis. Mechanistically, this composite nanovaccine synergized with IR to trigger ferroptosis and ICD via the STAT1‐IRF1‐ACSL4 axis, thereby remodeling the TME and overcoming the limitations of conventional vaccines by enhancing antigen release. In vivo, triple therapy with the liposomal carrier, IR, and anti‐PD‐L1 achieves robust anti‐tumor activity with negligible systemic toxicity and overcomes immune checkpoint blockade resistance. This study develops a translatable radiotherapy‐initiated in situ nanovaccination strategy relying on synergistic ferroptosis and ICD to treat refractory PAAD.

## Graphical abstract

Machine‐learning‐driven transcriptomic analysis identifies FSTL3 as a key tumor‐microenvironment regulator in desmoplastic pancreatic adenocarcinoma. A cRGD‐modified liposome co‐loading gemcitabine and siFSTL3 serves as an irradiation‐triggered in situ nanovaccine. It synergizes with radiotherapy to induce ferroptosis and immunogenic cell death through the STAT1‐IRF1‐ACSL4 axis, remodels the immunosuppressive TME, and restores anti‐PD‐L1 efficacy. This triple‐therapy strategy yields potent antitumor activity against refractory PAAD.

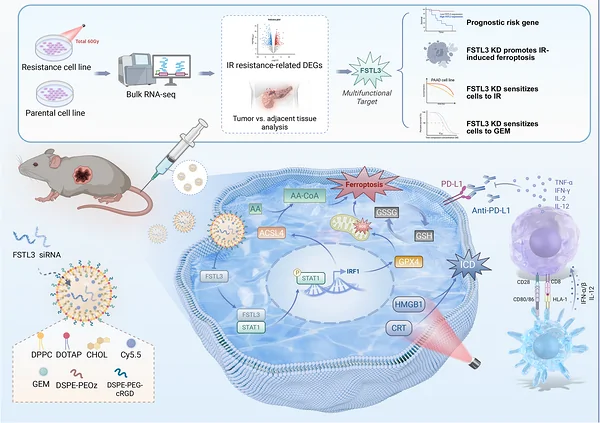

## 1 Introduction

Pancreatic adenocarcinoma (PAAD) is one of the most malignant tumors of the digestive system. It has a 5‐year survival rate of less than 10%. This low survival rate presents formidable challenges to clinical management [1, 2]. Radiotherapy is a pivotal approach for local tumor control. It damages the DNA of tumor cells through ionizing radiation, inducing apoptosis. However, using radiotherapy alone is often limited by the dense stromal barrier and immunosuppressive tumor microenvironment (TME) in PAAD. These factors frequently lead to radioresistance and tumor recurrence [3, 4]. Conventional immunotherapy also shows extremely low clinical response rates in PAAD. This is due to insufficient tumor antigen presentation efficiency and poor infiltration of effector T cells [5]. Therefore, developing combination therapies that synergistically reverse radioresistance and remodel the tumor immune microenvironment is a core focus in PAAD research.

The discovery of immunogenic cell death (ICD) has provided a key breakthrough for the synergy between radiotherapy and immunotherapy. Radiotherapy can not only directly eliminate tumor cells but also induce them to release tumor‐specific antigens (TSAs) and damage‐associated molecular patterns (DAMPs) such as high‐mobility group box 1 (HMGB1) and adenosine triphosphate (ATP). These factors can recruit antigen‐presenting cells (APCs), including dendritic cells (DCs), activate the body's specific anti‐tumor immune response, and form a “radiotherapy‐enhanced immunity” cascade reaction [6]. However, the ICD effect induced by radiotherapy monotherapy is weak, which is insufficient to break through the immunosuppressive TME of PAAD and fails to address radioresistance caused by tumor cell ferroptosis escape [7, 8].

Conventional therapeutic cancer vaccines, including nanovaccines, can induce potent anti‐tumor T cell responses by delivering tumor antigens and immunoadjuvants [9]. Tumor neoantigens derived from somatic mutations possess the unique property of being specifically expressed only on the surface of tumor cells, which not only provides a critical premise for evading central tolerance of T cells to self‐epitopes but also effectively elicits tumor‐specific T cell responses, thereby achieving precise elimination of tumor cells [10, 11]. However, tumors have developed multiple mechanisms to impair antigen presentation, such as causing antigen depletion and reduced surface expression of HLA‐I/MHC‐I through transcriptional regulation and genetic alterations [12, 13, 14]. Thus, there is an urgent need to develop novel design strategies for cancer vaccines.

Notably, lipid‐based nanomaterials, particularly those with a phospholipid bilayer structure, have emerged as highly promising drug delivery carriers. Such materials not only exhibit excellent biocompatibility but also enable the co‐encapsulation of multiple therapeutic agents and achieve precise drug controlled‐release effects [15, 16, 17]. Meanwhile, cationic liposomes themselves can serve as efficient delivery vectors for nucleic acid‐based agents and possess inherent adjuvant activity, which can synergistically enhance anti‐tumor immune responses [18]. This unique combination of properties makes lipid‐based nanomaterials ideal candidates for addressing the aforementioned challenges in cancer vaccine design, bridging the gap between drug delivery efficiency and immunomodulatory efficacy.

**SCHEME 1:**
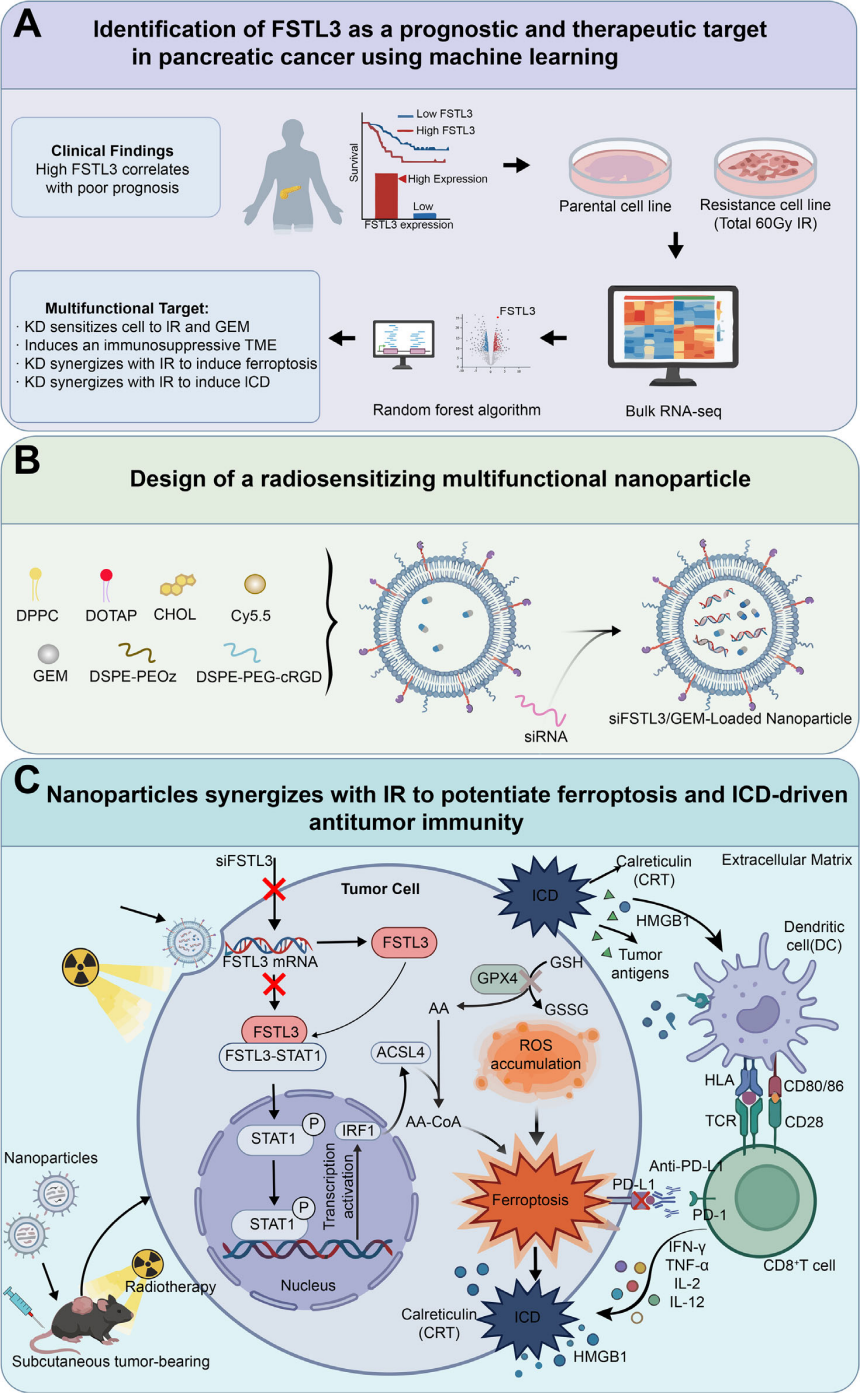
Schematic illustration of multifunctional target validation, nanovaccine synthesis, and their synergy with IR to induce multi‑cascade cell death for enhancing anti‑tumor immunity in pancreatic adenocarcinoma (PAAD). (A) Identification of FSTL3 as a prognostic and therapeutic target in PAAD using machine learning‐driven transcriptomic analysis. (B) Design of a radiosensitizing multifunctional nanovaccine. (C) Nanovaccine synergizes with ionizing radiation (IR) to potentiate ferroptosis and immunogenic cell death (ICD)‐driven antitumor immunity.

In summary, this work develops an irradiation‐activated in situ nanovaccination platform for PAAD based on cRGD‐functionalized liposomes combined with radiotherapy, targeting ferroptosis and TME remodeling. Different from conventional nanovaccines preloaded with exogenous antigens and adjuvants, our liposome co‐delivers gemcitabine and FSTL3 silencing payloads to induce in situ vaccination upon radiation. FSTL3, a prognostic target highly expressed in PAAD, when silenced, improves tumor chemo‐radiosensitivity, facilitates dendritic cell maturation, and reverses TME immunosuppression. Radiotherapy cooperates with liposomal gemcitabine to kill tumor cells and trigger ferroptotic ICD, releasing endogenous tumor antigens and DAMPs that function as intrinsic antigen and natural adjuvant, respectively. Meanwhile, FSTL3 knockdown amplifies radiotherapy‐mediated immune response, restores intratumoral immune infiltration and augments ferroptosis, creating a synergistic therapeutic loop. This study elucidates the synergistic molecular mechanism of the combined regimen, validates its potent anti‐tumor effects, and offers a novel translatable nanotherapeutic strategy for desmoplastic PAAD. The schematic illustration is presented in Scheme 1.

## 2 Results

### 2.1 Machine Learning‐Driven Transcriptomic Analysis Identified FSTL3 as a Key Target for the Development of Synthetic Radiotherapy‐Sensitizing Vaccines in PAAD

**FIGURE 1:**
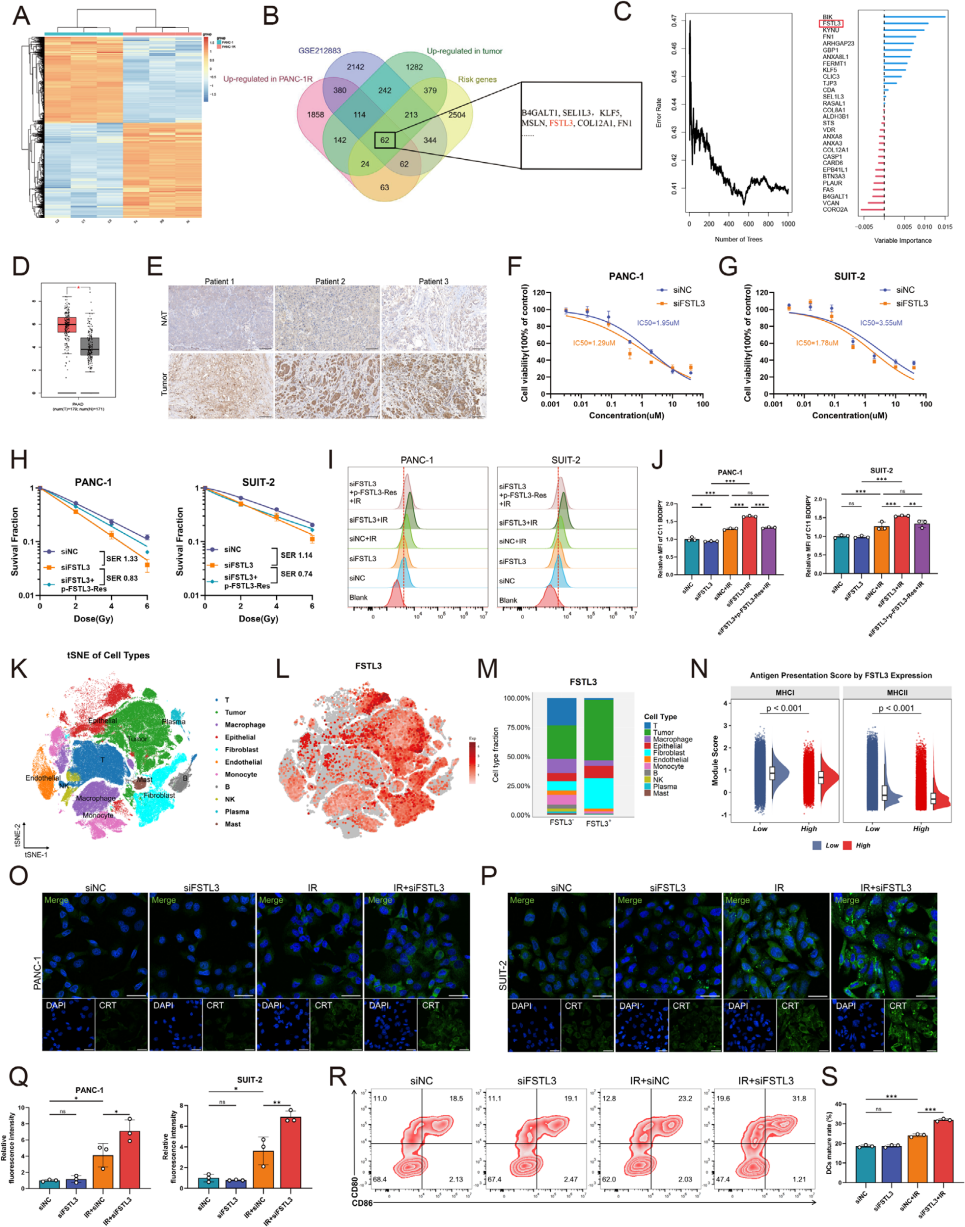
Machine learning‐driven transcriptomic analysis identified FSTL3 as a key target for designing radiotherapy‐sensitizing nanovaccine against PAAD. (A) Heatmap of differentially expressed genes (DEGs) identified by RNA‐seq in radioresistant PANC‐1R cells and parental PANC‐1 cells. (B) Venn diagram showing the intersection of DEGs upregulated in PANC‐1R cells, overexpressed in PAAD tissues, associated with poor prognosis, and linked to radioresistance in the GSE212883 cohort, yielding 62 candidate key molecules involved in PAAD progression. (C) Important genes associated with PAAD prognosis identified by random forest algorithm. (D) Box plots of FSTL3 expression in normal and tumor samples from the GEPIA database. (E) Immunohistochemical staining to evaluate FSTL3 protein abundance within tumor tissues and paired non‐neoplastic adjacent tissues of PAAD patients. (F, G) The IC50 of Gemcitabine (GEM) in PANC‐1 (F) and SUIT‐2 (G) cells was calculated from the drug inhibition curve. (H) Dose‐response analysis of survival fraction in PANC‐1 and SUIT‐2 cells with siNC, siFSTL3, or FSTL3 rescue upon 0–6 Gy radiation. SER: sensitizer enhancement ratio. (I) Flow cytometry histograms of C11‐BODIPY lipid peroxidation signals in PANC‐1 and SUIT‐2 cells under distinct treatment regimens. (J) Statistical quantification of C11‐BODIPY relative MFI. p‐FSTL3‐Res: siRNA‐resistant FSTL3 rescue plasmid. (K) TSNE plot of the PAAD scRNA‐seq data annotated by major cell types in the integrated cohort. (L) Expression levels of FSTL3 in cells illustrated in TSNE plots from the PAAD scRNA‐seq data. (M) Comparative analysis of cellular composition reveals that the FSTL3‐negative group has a markedly higher proportion of T cells, macrophages, and monocytes in PAAD tumor tissues compared to the FSTL3‐positive group. (N) Violin plots overlaid with boxplots showing module score distributions for MHCI and MHCII antigen presentation pathways in FSTL3‐low (blue) vs. FSTL3‐high (red) single cells (median split). (O, P) Representative immunofluorescence images depicting calreticulin (CRT) expression of PANC‐1 (O) and SUIT‐2 (P) cells under different treatment conditions. Scale bars, 50 µm. (Q) Quantitative analysis of CRT expression levels in PANC‐1 and SUIT‐2 cells across treatment groups. (R) Schematic illustration of the coculture system for PAAD mice PANC02 cells, and BMDCs. (S) Representative FCM plots showing changes in the proportion of CD80+CD86+ DCs under the indicated treatments. ^*^ *p* < 0.05, ^**^ *p* < 0.01, ^***^ *p* < 0.001.

After repeated fractional IR weekly with a total dose of 60 Gy, a radioresistant PAAD cell line, PANC‐1R, was constructed from the parental PANC‐1 cell line, with a sensitivity enhancement ratio (SER) of 1.52 (Figure S1A, B). To identify potential genes responsible for radiation resistance in pancreatic cancer cell lines, we conducted RNA‐seq analysis to investigate the mRNA expression profiles of the radiation‐resistant cell line (PANC‐1R) and its parental counterpart. A total of 9603 differentially expressed genes (DEGs) were identified, among which 2706 genes were significantly upregulated in the PANC‐1R cell line (Figure 1A). To further narrow down the candidate genes, we took the intersection of these upregulated genes with three sets of genes, namely: the genes that were significantly upregulated in radiation‐resistant cell lines from the GSE212883 cohort, prognosis‐related risk genes, and genes that were upregulated in tumor tissues. Ultimately, we found that there were 62 genes consistently upregulated across all these gene sets (Figure 1B). We further applied the random forest algorithm to screen prognosis‐related key genes in pancreatic cancer, with FSTL3 identified as the second most important gene (Figure 1C). The increased expression levels of FSTL3 are significantly correlated with shorter overall survival (OS), progression‐free survival (PFS), disease‐free survival (DFS), and relapse‐free survival (RFS) in these patients (Figure S2A–E). FSTL3 is highly expressed in tumor tissues of pancreatic cancer patients (Figure 1D, E; Figure S2F). To verify whether FSTL3 can affect the radio‐chemosensitivity of pancreatic cancer cells, we knocked down FSTL3 expression in PANC‐1 and SUIT‐2 cells using small interfering RNA (siRNA) (Figure S3A, B; Table S1). Cytotoxicity assays demonstrated that FSTL3 knockdown significantly impaired the proliferative activity of both cell lines (*p* < 0.05); moreover, when combined with radiotherapy, the cellular viability of these cells decreased further compared with that of cells treated with radiotherapy alone (*p* < 0.05). Notably, this enhanced radiosensitization effect triggered by FSTL3 silencing was fully rescued by ectopic overexpression of FSTL3 plasmid, restoring cell viability to levels comparable to the siNC+IR group (Figure S4A, B). To investigate the impact of FSTL3 on gemcitabine efficacy, we calculated the half‐maximal inhibitory concentration (IC50) of gemcitabine before and after FSTL3 knockdown. The results showed that the IC50 values of gemcitabine decreased from 1.95 to 1.29 µM in PANC‐1 cells (Figure 1F) and from 3.55 to 1.78 µM in SUIT‐2 cells (Figure 1G), demonstrating that FSTL3 silencing markedly sensitized pancreatic cancer cells to gemcitabine. Figure S5A, B displayed gemcitabine dose–response curves of the rescue group (siFSTL3+p‐FSTL3‐Res), and quantitative IC50 statistics in Figure S5C, D further verified that restoring FSTL3 expression fully reversed the reduced gemcitabine IC50 induced by FSTL3 knockdown. Knockdown of FSTL3 led to a significant decrease in the survival rate of PANC‐1 and SUIT‐2 cells following exposure to ionizing radiation (IR), with their SER reaching 1.33 and 1.14, respectively. Conversely, ectopic expression of FSTL3 fully rescued the enhanced radiosensitivity triggered by siFSTL3, with SER values restored to 0.83 and 0.74 in PANC‐1 and SUIT‐2 cells, respectively. These findings indicate that FSTL3 knockdown can sensitize pancreatic cancer cells to ionizing radiation (Figure 1H).

Ferroptosis is a unique form of regulated cell death characterized by iron‐dependent lipid peroxidation, a process regulated by multiple cellular metabolic pathways [19, 20]. Ferroptosis is closely correlated with radiosensitivity: ferroptosis‐resistant cancer cells contribute to radioresistance, whereas the induction of ferroptosis can enhance the radiosensitivity of cancer cells to radiotherapy [21]. We therefore investigated whether FSTL3 knockdown could synergize with radiotherapy to induce ferroptosis. Flow cytometry assays demonstrated that FSTL3 knockdown combined with radiotherapy resulted in a marked elevation of lipid peroxidation levels, while overexpressing FSTL3 fully reversed this phenotype (Figure 1I, J). These findings indicate that FSTL3 is a potential target for ferroptosis induction, which can exert a synergistic effect with radiotherapy to facilitate ferroptosis.

We further analyzed single‐cell sequencing data of pancreatic cancer from the integrated cohort, which revealed that FSTL3 was predominantly highly expressed in tumor cells and fibroblasts (Figure 1K, L). We further stratified the cell populations into two subsets based on FSTL3 expression levels: FSTL3^−^ and FSTL3^+^. Among FSTL3^+^ cells, tumor cells, fibroblasts, and epithelial cells accounted for a higher proportion, whereas FSTL3^−^ cells exhibited an increased proportion of T cells, macrophages, and monocyte cells (Figure 1M). Single‐cell transcriptomic analyses revealed that low FSTL3 expression was significantly associated with elevated module scores for both MHCI and MHCII antigen presentation pathways (Figure 1N; *p* < 0.001), indicating that FSTL3 downregulation correlates with enhanced antigen presentation capacity in the TME. The heatmap depicts the expression profiles of marker genes across 11 distinct cell subsets (Figure S6). Collectively, these results suggest that high FSTL3 expression may be associated with the suppressive tumor immune microenvironment (TIME) in PAAD and compromises antigen presentation capacity. ICD is a unique form of cell death wherein tumor cells, upon exposure to external stimuli, undergo a transition from non‐immunogenic to immunogenic, thereby mediating the body's anti‐tumor immune response [22, 23]. It can be triggered by various therapeutic modalities, including chemotherapeutic agents, radiotherapy, oncolytic viruses, and photodynamic therapy [24, 25, 26]. Although radiotherapy is capable of inducing ICD, its effects are transient and weak. Pancreatic cancer harbors an immunosuppressive TME and is classified as a “cold tumor” that is refractory to immunotherapy. IF assay demonstrated that the multifunctional nanovaccine combined with IR significantly upregulated Calreticulin (CRT) expression in both cell lines (Figure 1O–Q). This indicated that FSTL3 serves as a promising therapeutic target, and its knockdown can synergize with radiotherapy to induce ICD in pancreatic cancer cells. To investigate the effect of ICD on the antigen‐presenting capacity of DCs, we established a co‐culture system of bone marrow‐derived dendritic cells (BMDCs) and PANC02 cells (Figure S7). The knockdown efficiency of FSTL3 in PANC02 cells has been validated (Figure S8). Flow cytometry assays demonstrated that BMDCs cultured in the supernatant of FSTL3‐knockdown PANC02 cells treated with IR exhibited markedly enhanced maturation (Figure 1R,S). These findings demonstrate that FSTL3 is a promising multifunctional therapeutic target, and its knockdown can synergize with IR to induce multi‐cascade cell death in cancer cells.

### 2.2 Construction and Comprehensive Characterization of the Multifunctional Nanovaccine

**FIGURE 2:**
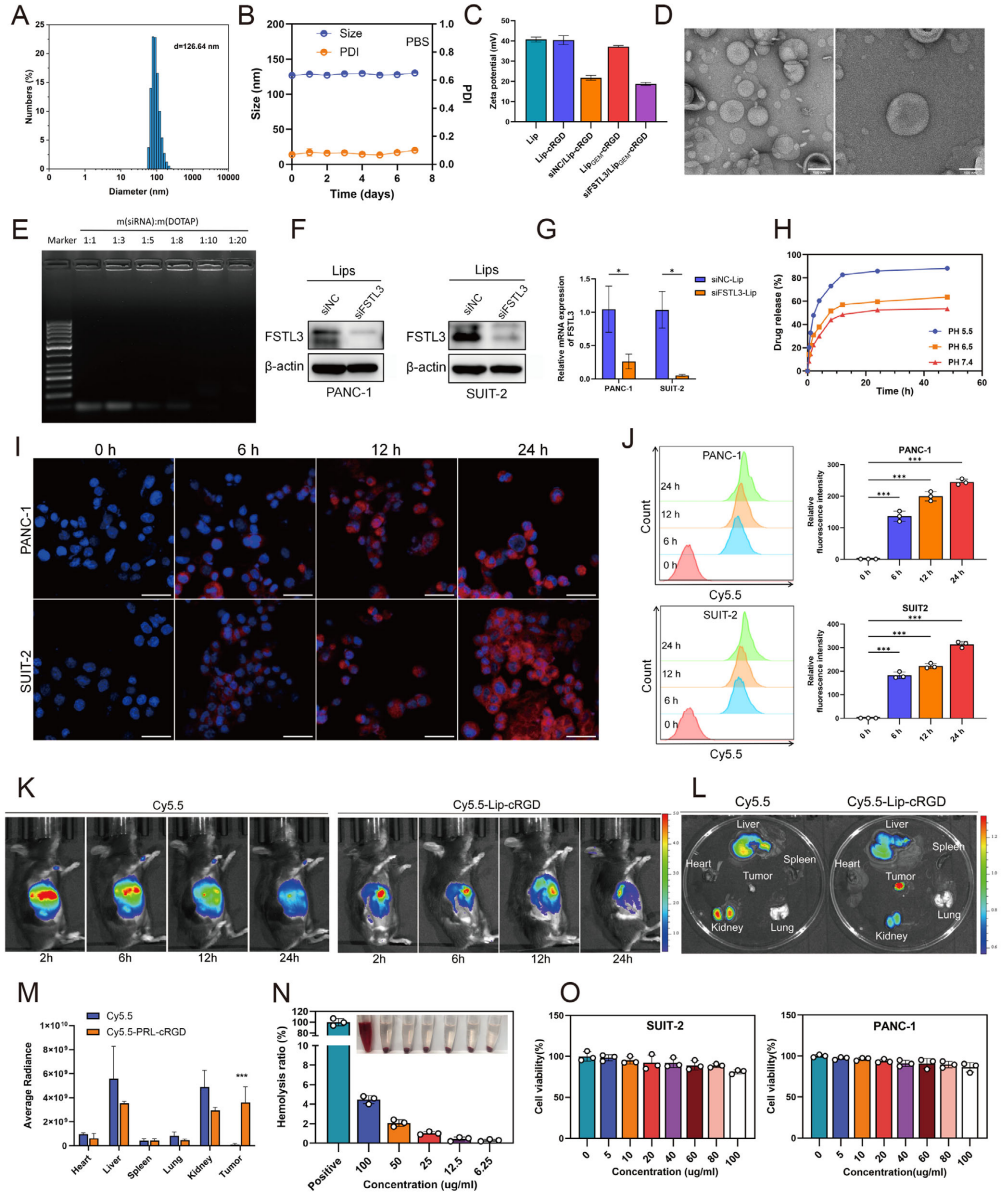
Synthesis and characterization of multifunctional nanovaccine (siFSTL3/Lip_GEM_‐cRGD). (A) Size distribution profile of siFSTL3/Lip_GEM_‐cRGD nanoparticles. (B) Changes in particle size and polydispersity index (PDI) for siFSTL3/Lip_GEM_‐cRGD incubated within PBS buffer. (C) Zeta‐potential measurements to compare surface electrostatic properties among various prepared formulations. (D) Representative transmission electron microscopy (TEM) images displaying the morphology of the composite nanovaccine. Scale bars, 100 nm. (E) Gel retardation assay evaluating siRNA binding capacity of cationic liposomes across varied mass ratios. (F) Western blot analyses indicating FSTL3 expression in PANC‐1 and SUIT‐2 cells treated with or without LNP‐encapsulated siFSTL3. (G) RT‐qPCR results indicating FSTL3 expression in PANC‐1 and SUIT‐2 cells treated with or without LNP‐encapsulated siFSTL3. (H) In vitro gemcitabine (GEM) release profiles measured under distinct pH environments (pH 5.5, 6.5, and 7.4). (I) Representative images of siNC/Lip‐cRGD uptake by PANC‐1 and SUIT‐2 cells at different time points (0, 6, 12, and 24 h). Scale bars, 50 µm. (J) Flow cytometry shows the quantitative analysis of nanovaccine uptake by PANC‐1 and SUIT‐2 cells at different time points (0, 6, 12, and 24 h). (K, L) Fluorescence imaging of in vivo (K) and ex vivo (L) distributions of nanovaccine in organs and tumors at different time points (0, 6, 12, and 24 h). (M) Quantitative analysis of nanovaccine distribution across different organs in the naked Cy5.5 group and Lip‐cRGD group (n = 3). (N) Hemolysis ratios induced by Lip‐cRGD at different concentrations and representative images. (O) Cytotoxicity of Lip‐cRGD in PANC‐1 and SUIT‐2 PAAD cell lines.

While FSTL3 knockdown markedly boosts the anti‐tumor efficacy of IR, the in vivo instability of the siFSTL3 plasmid severely restricts its potential for clinical translation. To address this limitation, a multifunctional nanovaccine directed against tumor cell‐intrinsic FSTL3 was developed to enhance therapeutic efficacy in a tumor‐specific manner. Furthermore, taking advantage of the distinctive property of liposomes to co‐encapsulate multiple therapeutic agents, we further encapsulated gemcitabine into these nanocarriers. Triplicate preparation experiments revealed stable encapsulation efficiency (EE) values ranging from 78.00% to 81.35%, with a mean encapsulation efficiency of approximately 79.28%, demonstrating reliable GEM loading performance of the nanocarrier (Table S5). This was designed to synergize with siFSTL3 and IR for augmented antigen production, thereby amplifying multi‐cascade cell death and further underscoring the potent anti‐tumor efficacy of this combinatorial regimen. DLS analysis revealed that the synthesized LNPs exhibited a uniform diameter with a normal distribution (Figure S9A–D), among which the particle size of siFSTL3/Lip_GEM_‐cRGD was approximately 126.64 nm (Figure 2A). After storing the nanovaccine in a 4°C refrigerator for one week, no significant changes were observed in its particle size or PDI, indicating that the nanovaccine could maintain a stable existence for a relatively long period (Figure 2B). Zeta potential measurements and TEM imaging revealing the structure of siFSTL3/Lip_GEM_‐cRGD were exhibited in Figure 2C,D. Agarose gel electrophoresis assays revealed that siRNA was nearly completely encapsulated into liposomes at a mass ratio of siRNA to DOTAP of 1:10 with an average EE of 92.22% (Figure 2E and Table S6). Results from RT‐qPCR and WB assays indicated that the nanovaccine we synthesized exhibited significant gene knockdown efficiency at both the mRNA and protein levels (Figure 2F,G). Figure 2H depicts the drug release profiles under simulated physiological (pH 7.4) and acidic (pH 6.5 and 5.5) conditions at 37°C. The results demonstrated that drug release was markedly enhanced in acidic environments; specifically, under the pH 5.5 condition that mimics the intracellular/lysosomal microenvironment, the cumulative release rate reached 88.27% ± 0.37% at 48 h. We assessed the cellular uptake of the nanovaccine by PANC‐1 and SUIT‐2 cells at 0, 6, 12, and 24 h post‐incubation. The results showed that the amount of nanovaccine internalized by the cells increased gradually over time, indicating that the nanovaccine possessed a favorable tumor cell‐targeting capacity (Figure 2I). Flow cytometry analysis further validated this conclusion (Figure 2J). In vitro and in vivo fluorescence imaging confirmed that compared with the injection of naked Cy5.5, Cy5.5 encapsulated by the nanovaccine was preferentially accumulated in tumor tissues, which directly verified the nanovaccine's robust tumor‐specific targeting capability (Figure 2K–M). The hemolysis assay demonstrated that the nanovaccine exhibited a hemolysis rate close to 0 at concentrations of 25 µg/mL and below, indicating favorable hemocompatibility of the nanovaccine. (Figure 2N) Cytotoxicity experiments revealed that elevating the concentration of empty Lip‐cRGD nanovaccine did not induce notable suppression of cell viability, verifying the excellent safety profile of the nanovaccine (Figure 2O).

### 2.3 Multifunctional Nanovaccine Acts Synergistically with IR to Markedly Boost both Tumor‐Suppressive Efficacy and Anti‐Tumor Immune Responses

**FIGURE 3:**
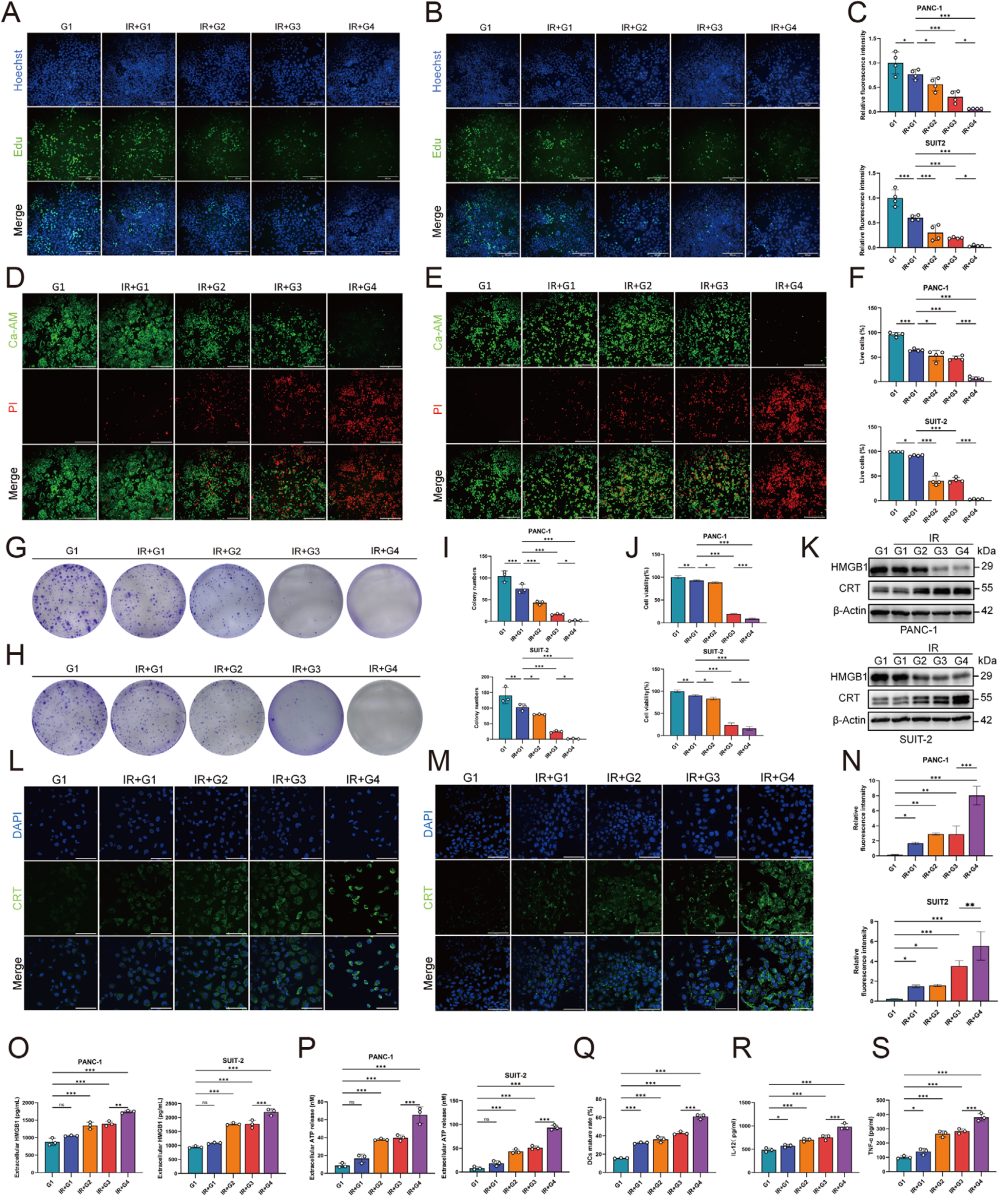
Multifunctional nanovaccine enhances the anti‐tumor efficacy of IR and anti‐tumor immune responses in PAAD cells. (A, B) G1, Lip‐cRGD (control); G2, siFSTL3/Lip‐cRGD; G3, siNC/Lip_GEM_‐cRGD; G4, siFSTL3/Lip_GEM_‐cRGD. EdU incorporation assay immunofluorescence staining to evaluate the proliferative capacity of PANC‐1 (A) and SUIT‐2 (B) cells under five different treatment conditions. (C) Quantification of EdU incorporation immunofluorescence of PANC‐1 and SUIT‐2 cells under five different treatment conditions (n = 4). (D, E) Live/dead staining assay of PANC‐1 (D) and SUIT‐2 (E) cells under five different treatment conditions. (F) Quantification of live/dead staining of PANC‐1 and SUIT‐2 cells under five different treatment conditions (n = 4). (G, H) Clonogenic assay to evaluate the proliferative capacity of PANC‐1 (G) and SUIT‐2 (H) cells under five different treatment conditions. (I) Quantification of clonogenic assay of PANC‐1 and SUIT‐2 cells under five different treatment conditions (n = 3). (J) CCK‐8 assay assessing the cell viability of PANC‐1 and SUIT‐2 PAAD cells. (K) Western blot analysis of HMGB1 and CRT expression in PANC‐1 and SUIT‐2 cells upon different treatments. β‑Actin served as the loading control. (L, M) Representative immunofluorescence images depicting calreticulin (CRT) expression of PANC‐1 (L) and SUIT‐2 (M) cells under different treatment conditions. Scale bars, 100 µm. (N) Quantitative analysis of CRT expression levels in PANC‐1 and SUIT‐2 cells across treatment groups. (O) ELISA quantification of extracellular HMGB1 concentrations in the culture supernatant of PANC‐1 and SUIT‐2 cells. (P) Luminescence‐based detection of extracellular ATP levels in the culture supernatant of PANC‐1 and SUIT‐2 cells. (Q) Quantification of BMDC maturation rate under different treatment groups. (R, S) The concentrations of IL‐12 (R) and TNF‐α (S) in the culture supernatants of differentially treated co‐cultures were determined by ELISA. ^*^ *p* < 0.05, ^**^ *p* < 0.01, ^***^ *p* < 0.001.

To assess the anti‐tumor efficacy of this multifunctional nanovaccine, we performed functional assays using two human PAAD cell lines, PANC‐1 and SUIT‐2. EdU assays demonstrated that a single‐loaded siFSTL3 nanovaccine inhibited tumor cell proliferation, while the dual‐loaded nanovaccine significantly potentiated this inhibitory effect on tumor cell proliferation (Figure 3A–C). Live/Dead cell viability staining further confirmed that LNPs‐mediated co‐delivery of gemcitabine and siFSTL3, in conjunction with IR, markedly elicited potent anti‐tumor effects while inducing a greater extent of tumor cell death (Figure 3D‐F). Consistently, the results of the colony formation assay and CCK‐8 confirmed that the combined delivery group (G4), in conjunction with IR, significantly suppressed the colony formation rate and proliferation of tumor cells (Figure 3G–J). Collectively, these results demonstrate that the multifunctional nanovaccine combined with IR significantly induce pancreatic cancer cell death.

CRT (Calreticulin) and HMGB1 (High‐mobility group box 1) are two critical classes of DAMPs released during ICD of tumor cells, serving as core biomarkers of ICD. Western blot analysis revealed that IR combined with the dual‐loaded nanovaccine markedly reduced HMGB1 levels, while substantially upregulating CRT expression in both PANC‐1 and SUIT‐2 pancreatic cancer cells (Figure 3K). The IF assay demonstrated that the multifunctional nanovaccine combined with IR significantly upregulated CRT expression in both cell lines (Figure 3L–N). ELISA and ATP quantification assays were performed to detect extracellular ICD markers HMGB1 (Figure 3O) and ATP (Figure 3P) in PANC‐1 and SUIT‐2 cells. IR combined with the dual‐loaded nanovaccine significantly enhanced HMGB1 and ATP secretion. This composite nanovaccine markedly promoted the release of HMGB1 and ATP. Collectively, these observations indicate that the composite nanovaccine is capable of amplifying ICD signals and boosting the antitumor activity induced by IR. To probe how ICD modulates the antigen‐presentation competence of dendritic cells, we constructed an in vitro co‐culture model consisting of bone marrow‐derived dendritic cells (BMDCs) and PANC02 tumor cells (Figure S10A). The knockdown efficiency of liposome‐encapsulated mouse siFSTL3 against FSTL3 at the protein level in PANC02 cells was validated (Figure S11). Flow cytometry results demonstrated that cells treated with dual‐loaded nanovaccine combined with IR significantly induced the maturation of BMDCs (Figure 3Q and Figure S10B). Concurrently, IR‐induced upregulated levels of anti‐tumor cytokines, including interleukin‐12 (IL‐12) and tumor necrosis factor‐α (TNF‐α), were significantly elevated after treatment with the nanovaccine (Figure 3R,S). Collectively, these findings underscore that the composite nanovaccine amplifies radiotherapy‐triggered ICD and initiates dendritic‐cell‐driven immune cascades, consequently strengthening antitumor therapeutic outcomes against PAAD.

### 2.4 Multifunctional Nanovaccine Enhances Ferroptosis Induced by IR in PAAD

**FIGURE 4:**
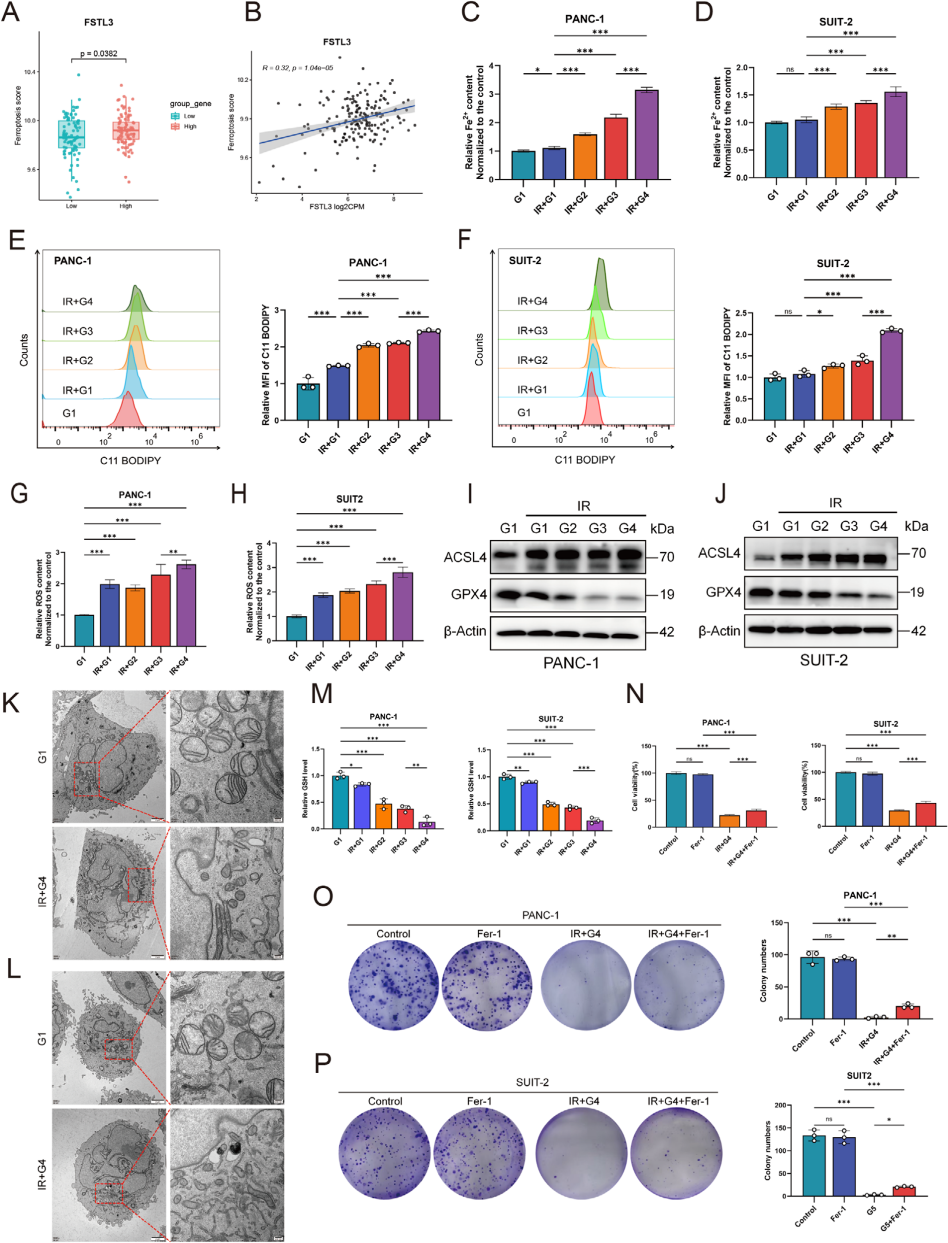
Multifunctional nanovaccine enhances IR‐mediated ferroptosis in PAAD cells. (A) Ferroptosis‐related scores for high‐FSTL3 and low‐FSTL3 subgroups within the TCGA‐PAAD patient cohort. (B) The relationship of FSTL3 expression with ferroptosis score. (C, D) Quantification of cellular iron contents in PANC‐1 (C) and SUIT‐2 (D) cells. (E, F) Flow cytometric analysis of lipid peroxidation levels in PANC‐1 (E) and SUIT‐2 (F) cells across five treatment groups, measured by C11 BODIPY fluorescence intensity, with the G1 group serving as the control. (G, H) Quantitative analysis of intracellular reactive oxygen species (ROS) levels in PANC‐1 (G) and SUIT‐2 (H) cells based on DCFDA fluorescence intensity; the G1 group was used as the control group. (I, J) Western blot analysis examining ACSL4 and GPX4 expression levels in PANC‐1 (I) and SUIT‐2 (J) cells under the indicated treatments. (K, L) Exemplary TEM micrographs depicting mitochondrial ultrastructural perturbations within PANC‐1 (K) and SUIT‐2 (L) cells upon combined irradiation (IR) plus composite nanovaccine administration. Low magnification (2 µm scale bar) and high magnification (200 nm scale bar) micrographs of boxed intracellular areas show intact mitochondrial cristae in the G1 group, while IR+G4 treatment induces prominent mitochondrial swelling, cristae disruption and vacuolization. (M) Quantitative analysis of intracellular GSH levels in PANC‐1 and SUIT‐2 cells. (N) CCK‐8 cytotoxicity assay was utilized to evaluate how co‐administration of Fer‐1 together with IR+G4 modulates the proliferative potential of PAAD tumor cells. (O, P) Clonogenic survival assays were performed to determine the influences of Fer‐1 plus IR+G4 combinatorial intervention on the growth potential of PANC‐1 (O) and SUIT‐2 (P) cells. ^*^ *p* < 0.05, ^**^ *p* < 0.01, ^***^ *p* < 0.001.

We evaluated the ferroptosis scores of pancreatic cancer patients in the TCGA dataset using the GSVA method. To further comprehensively assess ferroptosis in tumor cells following nanovaccine administration, we conducted a series of in vitro experiments. Results showed that patients with high FSTL3 expression exhibited a higher ferroptosis score (*p* < 0.05), and correlation analysis revealed that FSTL3 expression levels were significantly positively correlated with ferroptosis scores (R = 0.32, *p* < 0.05) (Figure 4A,B). Following treatment with dual‐loaded nanovaccine, tumor cells exhibited a significant increase in intracellular ferrous ion concentration (Figure 4C,D) and lipid peroxidation levels (Figure 4E,F). Consistently, intracellular reactive oxygen species (ROS) levels in tumor cells were significantly elevated following treatment with dual‐loaded nanovaccine (Figure 4G,H). Western blot analysis results demonstrated that following treatment with dual‐loaded nanovaccine, acyl‐coa synthetase long‐chain family member 4 (ACSL4) expression was significantly upregulated, while glutathione peroxidase 4 (GPX4) expression was remarkably downregulated in both PANC‐1 and SUIT‐2 cells (Figure 4I,J). TEM results demonstrated that in cells treated with IR combined with dual‐loaded nanovaccine, mitochondrial structures exhibited significant abnormalities, including decreased cristae, ruptured inner membranes, and mitochondrial shrinkage (Figure 4K,L). Moreover, intracellular glutathione (GSH) contents were markedly diminished within these cells (Figure 4M).

To verify the specificity of dual‐loaded nanovaccine in promoting ferroptosis, we performed a rescue assay using the ferroptosis inhibitor Ferrostatin‐1 (Fer‐1). CCK‐8 assay results consistently confirmed that the addition of Fer‐1 partially restored tumor cell viability following treatment with dual‐loaded nanovaccine combined with IR (Figure 4N). Colony formation assay results demonstrated that tumor cell colony formation was significantly suppressed following treatment with dual‐loaded nanovaccine combined with IR, while colony‐forming capacity was notably restored upon the addition of Fer‐1 (Figure 4O,P). These findings demonstrate that this composite nanovaccine potentiates IR‐induced ferroptosis, thereby amplifying the overall anti‐tumor efficacy.

### 2.5 Multifunctional Nanovaccine Promotes Ferroptosis in PAAD by Upregulating the STAT1‐IRF1‐ACSL4 Axis

**FIGURE 5:**
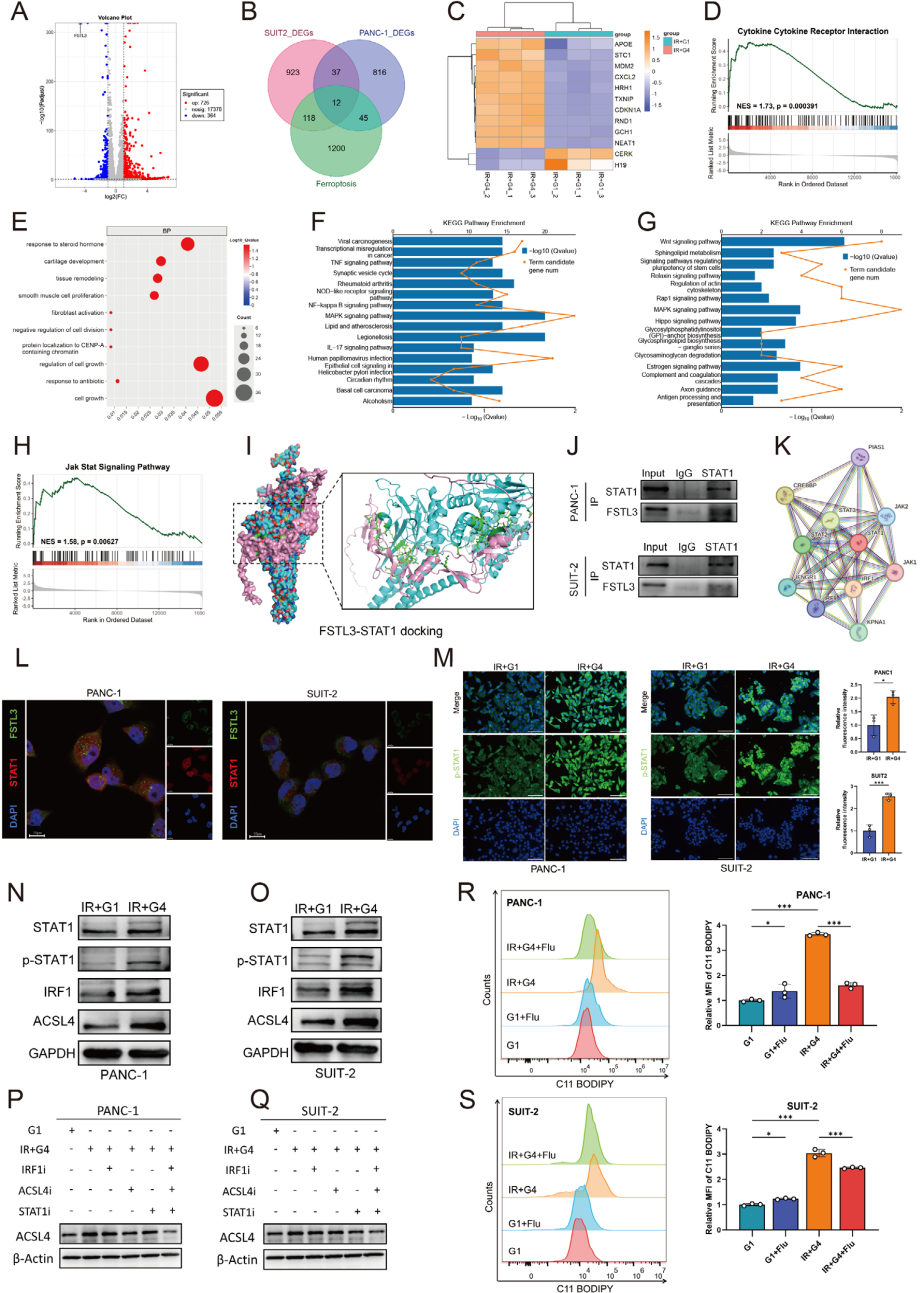
Multifunctional nanovaccine enhances PAAD ferroptosis via upregulating IRF1 and ACSL4 expression. (A) Volcano plot visualizing gene expression alterations following IR with and without FSTL3 perturbation in SUIT‐2 cells. (B) Venn diagram displaying the overlapping candidates between DEGs from siFSTL3/Lip_GEM_‐cRGD treated PANC‐1 and SUIT‐2 cells and known ferroptosis‐associated gene sets. (C) Heatmap visualizing transcriptional profiles of 12 representative ferroptosis‐relevant genes in SUIT‐2 cells. (D) Gene set enrichment analysis (GSEA) demonstrating IR‐associated cytokine–cytokine receptor interactions. (E) GO analysis was performed in the aspect of biological processes (BP). (F) Kyoto Encyclopedia of Genes and Genomes (KEGG) pathway analysis. The orange curve represents the number of candidate genes. (G) KEGG pathway analysis of down‐regulated differentially expressed genes. (H) GSEA results demonstrating the enrichment of the IR‐associated JAK‐STAT signaling pathway. (I) Molecular docking simulations illustrating the interaction between FSTL3 and STAT1. (J) Co‐immunoprecipitation (Co‐IP) assays verifying the endogenous binding between FSTL3 and STAT1 in PANC‐1 and SUIT‐2 pancreatic cancer cell lines. Cell lysates were immunoprecipitated with anti‐STAT1 antibody or control IgG, followed by immunoblotting against FSTL3 and STAT1. Input represents total protein lysate without immunoprecipitation. (K) Protein‐protein interaction (PPI) network analysis displaying STAT1‐centered interacting partners involved in interferon and JAK‐STAT signaling cascades. (L) Representative confocal microscopy images of FSTL3 (green), STAT1 (red), and DAPI‐stained nuclei (blue) in PANC‐1 and SUIT‐2 cells. Merged channels show prominent overlapping punctate signals of FSTL3 and STAT1 in the cytoplasmic compartment of both pancreatic cancer cell lines. Scale bars, 10 µm. (M) Representative images of immunofluorescence staining and quantitative analysis showing p‐STAT1 protein levels following IR+G4 treatment in PANC‐1 and SUIT‐2 cells. Scale bars, 100 µm. (N, O) Western blot analysis of phosphorylated STAT1 (p‐STAT1), STAT1, IRF1, ACSL4, and actin expression following IR+G4 treatment in PANC‐1 (N) and SUIT‐2 (O) cells. (P, Q) Western blot showing ACSL4 levels in PANC‐1 and SUIT‐2 cells. Treatments: G1 control; IR+G4±IRF1i (IRF1‐IN‐1), ACSL4i (LIBX‐A401), STAT1i (fludarabine) single or triple inhibitors. (R, S) Flow cytometric analysis of lipid peroxidation levels in PANC‐1 (R) and SUIT‐2 (S) cells treated with IR+G4 and/ or STAT1 inhibitor fludarabine. ^*^ *p* < 0.05, ^**^ *p* < 0.01, ^***^ *p* < 0.001.

To further investigate the molecular regulatory cascades driving the synergistic actions of composite nanovaccine combined with IR in PAAD, we carried out transcriptome profiling for cell‐derived samples collected from the IR and IR+G4 experimental cohorts. Volcano plots highlighted DEGs after the composite nanovaccine treatment (Figure 5A; Figure S12A). By performing overlap analysis between DEGs and ferroptosis‐related genes, we identified twelve genes that may play key roles in ferroptosis induction by this system. Among these genes, APOE, STC1, MDM2, CXCL2, HRH1, TXNIP, CDKN1A, RND1, GCH1, and NEAT1 were upregulated in the IR+G4 group, while CERK and H19 were downregulated (Figure 5B,C). The transcriptional alterations of these genes within IR+G1 and IR+G4 cohorts recapitulate typical ferroptosis‐related signatures, which supports the notion that this multifunctional nanovaccine boosts radiosensitivity and antitumor potency via triggering ferroptosis. Gene Set Enrichment Analysis (GSEA) identified enriched signaling modules linked to cytokine‐receptor crosstalk (Figure 5D). 3D GO annotation (CC, BP, MF) further demonstrated that DEGs were predominantly enriched in biological events relevant to tumor cellular phenotypes and oncogenic growth, encompassing cell division inhibition, fibroblast activation, as well as DNA‐binding transcription activator function (Figure 5E; Figure S12B, C). KEGG pathway analysis highlighted the enriched DEGs in anti‐tumor immune‐related pathways, including the TNF signaling pathway and IL‐17 signaling pathway. We further conducted KEGG pathway enrichment analysis on the downregulated DEGs, and the results revealed that the ferroptosis‐related sphingolipid metabolism pathway and the immune activation‐associated antigen processing and presentation pathway were highly enriched (Figure 5F,G). GSEA also indicated a strong association between IR+G4 treatment and upregulation of the JAK‐STAT pathway (Figure 5H). Molecular docking simulations suggested a potential interaction between FSTL3 and STAT1 (Figure 5I), which was further validated by immunoprotein assays (Figure 5J). Protein‐Protein Interaction (PPI) network analysis and correlation analysis revealed that STAT1 and IRF1 exhibited a strong positive correlation (R = 0.59, *p* < 0.05) (Figure 5K; Figure S13). Immunofluorescence confocal microscopy was applied to characterize the subcellular distribution of FSTL3 and STAT1 in pancreatic cancer cells. As shown in merged images, abundant overlapping punctate fluorescence signals of FSTL3 and STAT1 were observed within the cytoplasm of both PANC‐1 and SUIT‐2 cell lines, demonstrating obvious spatial co‐localization of the two proteins (Figure 5L). Consistently, western blot analysis verified that FSTL3 functionally modulates STAT1 phosphorylation under radiation stress in PANC‐1 pancreatic cancer cells (Figure S14A,B). Nanovaccine treatment significantly boosted irradiation‐stimulated p‐STAT1 expression, and this inductive effect was substantially suppressed upon exogenous FSTL3 overexpression. This finding indicates that the multifunctional nanovaccine may promote ferroptosis through the STAT1‐IRF1 pathway. IF staining results demonstrated that the protein level of p‐STAT1 was significantly upregulated both in PANC‐1 and SUIT‐2 cells treated with dual‐loaded nanovaccine (Figure 5M). Western blot analysis demonstrated that the expression levels of phosphorylated STAT1 (p‐STAT1), IRF1, and ACSL4 were significantly upregulated in PANC‐1 and SUIT2 cells following combined treatment with siFSTL3/Lip_GEM_‐cRGD and IR (Figure 5N,O; Figure S15A,B). To validate the critical role of the STAT1‐IRF1‐ACSL4 axis in FSTL3‐mediated ferroptosis activation, we treated cells with fludarabine (a specific STAT1 inhibitor), IRF1‐IN‐1 (a specific IRF‐1 inhibitor) or LIBX‐A401 (a specific ACSL4 inhibitor). Western blot analysis demonstrated that these inhibitors effectively inhibit the expression of ACSL4 respectively (Figure 5P,Q). Moreover, The results of flow cytometric analyses showed that these inhibitors effectively inhibited ferroptosis activity induced by dual‐loaded nanovaccine and significantly reduced lipid peroxidation levels (Figure 5R,S; Figure S16A–H).

### 2.6 Multifunctional Nanovaccine Potentiates Radiotherapy Against PAAD, Generates Memory‐Like Systemic Antitumor Immune Responses with Minimal Toxicity

**FIGURE 6:**
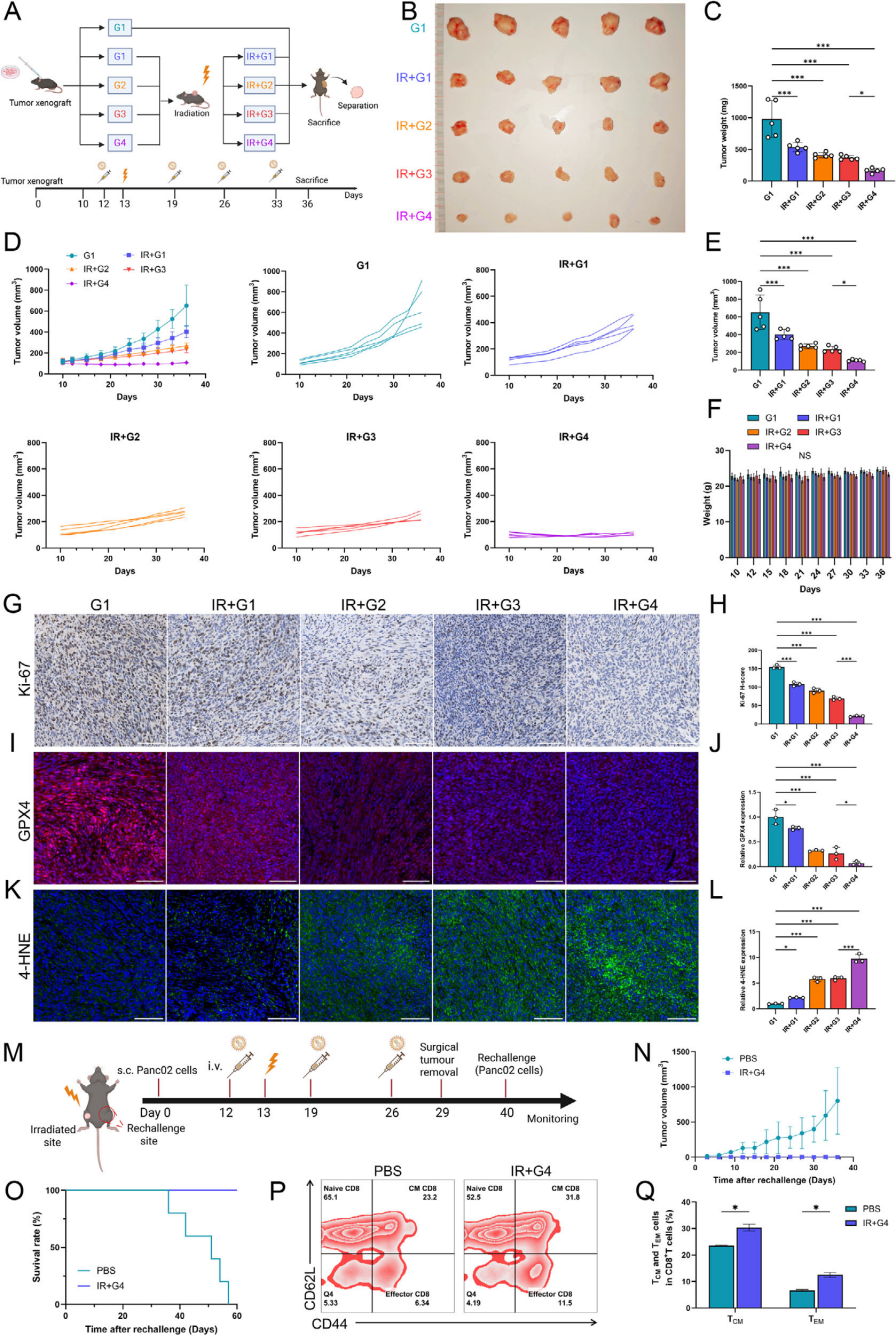
Multifunctional nanovaccine potentiates the anti‐tumor efficacy of IR in PAAD, induces memory‐like systemic anti‐tumor immune response to resist tumor rechallenge, and maintains a favorable safety profile. (A) Flowchart of the animal model illustrating the treatment timeline and nanovaccine administration following IR. (B) Variations in tumor volume across diverse treatment groups upon IR exposure (n = 5). (C) Histogram comparing tumor weights across treatment groups, demonstrating the lowest tumor weight in the IR + G4 nanovaccine group. (D) Time‐dependent tumor volume growth curves of each group and individual tumor growth trajectories of mice within respective cohorts. (E) Quantitative statistical analysis of final tumor volume at the endpoint. (F) Quantitative bar plot depicting mouse body‐weight variations throughout different therapeutic cohorts. (G) Representative immunohistochemical images of Ki‐67 and histochemistry score (H‐score) (H) in the tumor across treatment groups. Scale bars, 100 µm. (I) Representative immunofluorescence staining of GPX4 and quantitative analysis of protein expression (J) in the tumor across treatment groups. (K) Representative immunofluorescence staining of 4‐HNE and quantitative analysis of protein expression (L) in the tumor across treatment groups. (M) Schematic timeline of PANC02 tumor resection and rechallenge model. Mice were subcutaneously injected with PANC02 cells at day 0, treated with IR plus G4 nanovaccine, surgically resected of primary tumors on day 29, and rechallenged with PANC02 cells on day 40 to evaluate long‐term immune protection. (N) Tumor volume growth of PBS and IR+G4 groups after PANC02 rechallenge (n = 5). Data are presented as mean ± SD. (O) Kaplan–Meier survival curves of PBS‐ and IR+G4‐treated mice post tumor rechallenge (n = 5). (P) Representative flow cytometry contour plots of splenic CD8^+^ T subsets (central memory T, T_CM_; effector memory T, T_EM_) stained for CD44 and CD62L. (Q) Statistical quantification of T_CM_ and T_EM_ proportions within total CD8^+^ T cells (n = 3). Data are presented as mean ± SD; ^*^ *p* < 0.05, ^**^ *p* < 0.01, ^***^ *p* < 0.001.

In vitro experiments demonstrated that the multifunctional nanovaccine could significantly potentiate the anti‐tumor efficacy of IR. To further investigate its in vivo therapeutic efficacy, we designated Group 1 (G1) as the control group and Groups 2 to 4 (G2–G4) as the treatment groups. Following the successful establishment of the tumor‐bearing model, the first administration of the nanovaccine was performed on day 12, which was 1 day before radiotherapy; subsequently, IR was delivered on day 13, and additional nanovaccine treatments were administered on days 19, 26, and 33, respectively (Figure 6A). Compared with the control group, the treatment groups exhibited significant reductions in tumor volume, weight, and growth rate. nanovaccine loaded with gemcitabine alone or siFSTL3 alone showed comparable therapeutic efficacy, whereas nanovaccine co‐loaded with both agents achieved the most pronounced tumor suppressive effect (Figure 6B–E). Furthermore, no significant changes in body weight were observed across all groups (Figure 6F). Histological analysis demonstrated that no obvious structural alterations were detected in the tissues from each group (Figure S17). Compared with the control group, the other groups showed no significant differences in liver function indices (alanine aminotransferase [ALT], aspartate aminotransferase [AST], and albumin [ALB]), myocardial enzyme marker (creatine kinase [CK]), and renal function index (blood urea nitrogen [BUN]), indicating the excellent biocompatibility of IR combined with nanomaterials (Figure S18). HE‐stained sections of tumor tissues showed that tumor necrosis gradually increased from group G1 to the IR+G4 group, accompanied by a progressive reduction in Ki67 expression (Figure S17 and Figure 6G,H). These findings indicate that the addition of the composite nanovaccine significantly suppressed tumor growth. Consistent with our in vitro observations, IF analysis further validated that the composite nanovaccine treatment led to a significant reduction in GPX4 expression and a robust accumulation of 4‐hydroxynonenal (4‐HNE) within tumor tissues (Figure 6I–L). This phenotypic change directly evidenced the potent induction of ferroptosis in tumor tissues by the composite nanovaccine.

To verify whether nanovaccine generates long‐term antitumor immune memory, we constructed a pancreatic tumor rechallenge model after primary tumor resection (Figure 6M). Tumor volume monitoring revealed nearly complete suppression of secondary tumor outgrowth in the IR+G4 group, whereas robust tumor progression was observed in PBS‐treated mice following rechallenge (Figure 6N). Kaplan–Meier survival curves showed markedly extended survival in the IR+G4 group relative to PBS controls after tumor rechallenge (Figure 6O). Flow cytometry was further performed to characterize CD8^+^ memory T cell subsets using CD44 and CD62L markers. Representative contour plots and statistical quantification revealed significantly higher proportions of central memory T (T_CM_) and effector memory T (T_EM_) CD8^+^ cells in IR+G4‐treated mice (Figure 6P,Q). In summary, radiotherapy combined with nanovaccine elicits robust memory‐like CD8^+^ T‐cell responses, which provide systemic anti‐tumor activity against pancreatic tumor recurrence and secondary tumor engraftment.

### 2.7 Multifunctional Nanovaccine Potentiates Anti‐Tumor Immunity and Augment the Therapeutic Efficacy of Anti‐PD‐L1 Therapy

**FIGURE 7:**
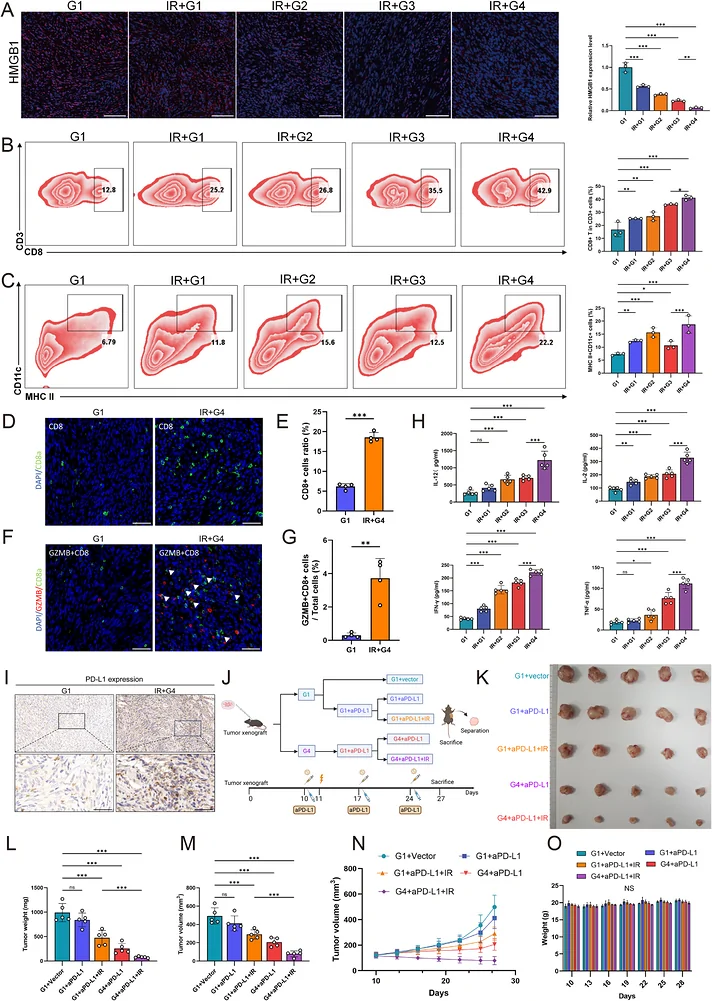
Multifunctional nanovaccine enhance IR‐induced anti‐tumor immune effects and anti‐PD‐L1 therapeutic efficacy. (A) Representative IF images presenting relative HMGB1 abundance in PAAD tumor specimens, paired with quantitative bar graphs for expression measurement (n  =  3). Scale bars, 100 µm. (B) False‐color flow cytometry dot‐plots exhibiting CD3 and CD8 profiles among live‐gated PAAD tumor cells, with bar charts summarizing CD3+CD8+ T‐cell proportions (n  =  3). (C) Flow cytometry false‐color plots depicting CD11c and MHC‐II expression in live‐cell gated PAAD tumor tissues, with histograms showing the percentage of CD11c+MHC‐II+ T cells (n = 3). (D‐E) Immunofluorescence micrographs visualizing CD8+ cells within PAAD tumor tissues, paired with quantitative analysis of CD8+ cell fractions relative to total cells. Scale bars, 50 µm. (F, G) Representative micrographs of CD8+ and GZMB+ cells in PAAD tumor tissues, together with quantitative quantification of GZMB+ cell proportions among the CD8+ population. (H) Serum ELISA measurements for multiple cytokines, including tumor necrosis factor‐alpha (TNF‐α), interleukin‐2 (IL‐2), interleukin‐12 (IL‐12), and interferon‑gamma (IFN‐γ), from mice receiving distinct therapeutic regimens. (I) PD‐L1 protein abundance was evaluated in PAAD tumor specimens derived from mice receiving IR combined with dual‐loaded nanovaccine and control counterparts. Scale bars, 50 µm. (J) Treatment scheme for PANC02 tumor‐bearing mouse models, illustrating the experimental timeline and interventions. (K) Comparative tumor‐size profiles among various anti‐PD‐L1‐combined treatment cohorts (n  =  5). (L) Bar graphs summarizing tumor‐weight data for all experimental groups. (M) Bar charts illustrating tumor‐volume statistics across cohorts. (N) Tumor‐growth kinetics curves for individual treatment groups. (O) Quantitative bar graphs monitoring body‐weight variations throughout treatment. ^*^ *p* < 0.05, ^**^ *p* < 0.01, ^***^ *p* < 0.001.

The antitumor immune effects of the multifunctional nanovaccine combined with IR were further validated in PAAD. Consistent with the in vitro experimental results, the co‐delivered nanovaccine significantly downregulated the expression of HMGB1, thereby enhancing the efficacy of IR‐induced ICD (Figure 7A). Flow cytometry assays revealed a marked increase in CD11c^+^MHCII^+^ DC cells and CD8^+^CD3^+^ T cells following dual‐loaded nanovaccine treatment, suggesting that the co‐delivered nanovaccine potently enhances anti‐tumor immune responses in the TME (Figure 7B,C). Specifically, the proportion of MHC‐II^+^ DCs increased significantly from 6.79% in the control group to 22.2% in the IR + G4 group, with statistically significant differences observed among consecutive treatment groups (Figure 7C). Similarly, the CD3^+^CD8^+^ T cell population expanded substantially from 12.8% in the control group to 42.9% in the IR + G4 group, indicating a marked enhancement in the recruitment and activation of T cells (Figure 7B). IF assays confirmed that dual‐loaded nanovaccine treatment significantly increased the numbers of GZMB^+^CD8^+^ double‐positive cells and CD8^+^ T cells (Figure 7D–G). In the IF images, white arrows highlighted a marked increase in CD8^+^ T cells and GZMB^+^CD8^+^ double‐positive cells in tumor tissues of the IR + G4 group compared with the G1 group. Furthermore, following treatment with co‐delivered nanovaccine, the levels of IR‐induced anti‐tumor cytokines—including interferon‐γ (IFN‐γ), interleukin‐2 (IL‐2), interleukin‐12 (IL‐12), and tumor necrosis factor‐α (TNF‐α)—were significantly upregulated (Figure 7H). These findings indicate that the co‐delivered nanovaccine can effectively activate and potentiate IR‐induced anti‐tumor immune responses in vivo, thereby enhancing the anti‐tumor efficacy.

Immunohistochemistry analyses showed obvious upregulation of PD‐L1 (Programmed Death‐Ligand 1) in comparison with control samples. These findings implied that PD‐L1 blockade is capable of boosting the antitumor performance of the co‐administered nanovaccine (Figure 7I; Figure S19). Therefore, we further combined anti‐PD‐L1 therapy with the nanovaccine to investigate the impact of the combination of the co‐delivered nanovaccine and IR on the therapeutic efficacy of anti‐PD‐L1 therapy (Figure 7J). In vivo experimental results demonstrated that the combination therapy of anti‐PD‐L1 antibodies and the co‐delivered nanovaccine could effectively inhibit the progression of PAAD (Figure 7K–N). Furthermore, this combination therapy exhibited favorable biocompatibility. No significant differences in body weight were observed among mice in all groups, and no notable alterations were detected in liver function markers (ALT, AST, and ALB) or renal function parameters (blood urea nitrogen, BUN) of the mice (Figure 7O; Figure S20). These results suggest that combining the multifunctional nanovaccine with IR and anti‐PD‑L1 monoclonal antibodies may improve the therapeutic performance of anti‐PD‐L1‑based immunotherapy, pointing toward a potential combinatorial strategy for PAAD treatment.

## 3 Discussion

Pancreatic cancer is an aggressive malignancy with an immunosuppressive cold TME and intrinsic dual resistance to radiotherapy and immunotherapy, leading to extremely poor clinical outcomes. However, pancreatic cancer frequently develops radiation resistance due to its suppressive TME and altered gene expression profiles, ultimately leading to treatment failure [27]. To break this bottleneck, we propose an innovative radiotherapy‐triggered in situ tumor vaccination strategy, which generates intrinsic tumor vaccines inside tumors after radiation exposure without loading exogenous antigens.

In this study, we performed machine learning screening combining radioresistant pancreatic cancer cell RNA‐seq and TCGA/GEO datasets, and identified FSTL3 as a key target to overcome PAAD radioresistance. FSTL3 overexpression in tumor tissues correlates with poor patient prognosis; FSTL3 silencing sensitizes cancer cells to IR and gemcitabine, and augments IR‐induced lipid peroxidation and ferroptosis. Single‐cell sequencing proved high FSTL3 suppresses antigen presentation and sustains an immunosuppressive TME, whereas FSTL3 knockdown robustly enhances IR‐triggered ICD and DCs maturation. The synchronous ferroptosis and ICD induced by IR plus FSTL3 inhibition release massive endogenous tumor antigens and DAMPs, which constitute the core antigen pool for radiotherapy‐activated in situ vaccination. Based on this mechanism, we constructed a pH‐sensitive cRGD‐modified liposomal nanovaccine (siFSTL3/Lip_GEM_‐cRGD) co‐delivering FSTL3 siRNA and gemcitabine to realize targeted intratumoral FSTL3 depletion. This nanoplatform greatly amplifies IR‐induced ferroptosis and ICD, continuously supplying tumor antigens within lesions and driving effective in situ vaccination to suppress tumor proliferation.

Mechanistically, the STAT1‐IRF1‐ACSL4 signaling axis dominates nanovaccine‐boosted ferroptosis and subsequent in situ vaccination. The nanocarrier blocks FSTL3–STAT1 binding to elevate STAT1 phosphorylation, upregulate IRF1 transcription and downstream ACSL4 expression that fuels lipid peroxidation. ACSL4 catalyzes the conjugation of free arachidonic acid and other long‐chain polyunsaturated fatty acids (PUFAs) with coenzyme A to generate the corresponding acyl‐CoA esters, which serve as substrates for lipid peroxidation [28]. Meanwhile, it downregulates GPX4 and depletes intracellular GSH to further accumulate lipid peroxides and strengthen ICD‐mediated antigen release. STAT1 inhibitor treatment completely abolished this cascade, confirming the axis is essential for supporting radiotherapy‐initiated in situ vaccination. Beyond direct radiation cytotoxicity, this signaling cascade remodels the suppressive TME and facilitates DC antigen presentation, a prerequisite for robust anti‐tumor immunity elicited by in situ vaccines.

Follistatin‐like 3 (FSTL3) is a secreted glycoprotein belonging to the follistatin‐module protein family, and its overexpression promotes tumor immune evasion and resistance to immunotherapy [29, 30]. Studies have demonstrated that FSTL3 knockout increases the proportion of CD8^+^T cells in the TME, reduces the proportions of regulatory T cells (CD25^+^Foxp3^+^) and exhausted T cells (PD1^+^CD8^+^), and synergistically enhances the therapeutic efficacy of anti‐PD‐1 immunotherapy [29]. Thus, FSTL3 represents a highly promising target for tumor immunotherapy. Besides, FSTL3 exhibits critical biological functions in tumor stromal cells and acts as a key regulator of stromal remodeling and immune suppression. Stromal FSTL3 derived from CAFs actively promotes extracellular matrix deposition and tumor fibrosis, facilitates epithelial‐mesenchymal transition, tumor invasion and metastasis, and shapes an immunosuppressive stromal microenvironment [31, 32]. Consistent with these previous findings, our single‐cell data demonstrate that FSTL3 expressed in tumor cells and CAFs shapes the cold TME: the FSTL3‐low subgroup harbors abundant infiltrating T cells, macrophages and monocytes capable of responding to antigens released by in situ vaccination. In vivo animal assays further verified that nanovaccine combined with IR elevates intratumoral MHCII^+^ DCs and cytotoxic GZMB^+^CD8^+^ T cells, demonstrating that FSTL3 silencing reshapes the fibrotic immunosuppressive TME and unlocks the full antitumor potential of radiotherapy‐activated in situ vaccines.

PD‐L1 is a crucial immune checkpoint molecule. Upon binding to PD‐1 on immune cells, it elicits a unique immune evasion microenvironment, which suppresses the proliferation and activation of CD4^+^ T lymphocytes and CD8^+^ T lymphocytes as well as their anti‐tumor immune responses. This impairs the cytotoxic activity of T lymphocytes against tumor cells, leading to immune evasion of malignant tumors and subsequent rapid tumor progression [33, 34]. Inhibition of PD‐L1 can enhance the cytotoxic and antigen‐recognition capabilities of host immune cells, and PD‐L1‐targeted inhibitors can significantly enhance the efficacy of tumor immunotherapy [35, 36]. Most subtypes of pancreatic cancer are classified as immunologically cold or immune‐resistant tumors, and the clinical efficacy and therapeutic responsiveness of PD‐1/PD‐L1 immune checkpoint inhibitor therapy for pancreatic cancer are highly dependent on the tumor immune microenvironment and PD‐L1 expression levels [37, 38]. Our nanovaccine upregulates tumor PD‐L1 upon IR exposure; importantly, the abundant self‐antigens produced by radiotherapy‐triggered in situ vaccination create a therapeutic window for checkpoint blockade combination. In vivo treatment validation showed that triple therapy (nanovaccine + IR + anti‐PD‐L1) confers potent tumor‑suppressive effects, potentially driven by combined benefits from FSTL3 silencing, chemotherapy, radiation‐mediated cytotoxicity, and immune checkpoint blockade. By contrast, anti‐PD‐L1 monotherapy yields negligible anti‐tumor activity, suggesting that radiotherapy‐triggered in situ antigen generation may serve as a critical prerequisite for overcoming immunotherapy resistance in PAAD [39].

This work develops a clinically translatable radiotherapy‐initiated in situ nanovaccination platform to overcome therapeutic resistance in refractory PAAD, though several limitations remain for follow‐up research. Mouse tumor models possess far less dense stromal fibrosis than human pancreatic cancer, which may overestimate nanocarrier penetration efficiency and the magnitude of in situ vaccine formation; further verification in patient‐derived organoids (PDOs) and patient‐derived xenografts (PDXs) is required. In the current study, we validated the formation of long‐term anti‐tumor immune memory via tumor rechallenge assays and memory T cell subset quantification. Besides, a CD8^+^ T cell depletion experiment is absent in our present dataset, which would be necessary to definitively demonstrate that the therapeutic efficacy truly depends on adaptive antitumor immunity. Subsequent research should systematically characterize the nanovaccine's long‐term in vivo immunomodulatory activity, biocompatibility and immunogenicity, as well as the long‐term biological risks caused by persistent FSTL3 knockdown, to advance the clinical translation of this radiation‑activated in situ vaccination strategy.

## 4 Conclusion

This study identifies FSTL3 via machine learning‐guided transcriptomic profiling and clarifies its dual role in PAAD as both a driver of radioresistance and a mediator of immunosuppression. We constructed a cRGD‐modified liposomal nanovaccine co‐loading gemcitabine and siFSTL3 (siFSTL3/Lip_GEM_‐cRGD) for synergistic radiotherapy. Unlike conventional antigen‐preloaded nanovaccines, this carrier enables radiotherapy‐activated in situ tumor vaccination: upon IR, targeted FSTL3 silencing activates the STAT1‐IRF1‐ACSL4 axis to trigger synchronous ferroptosis and ICD, releasing endogenous tumor antigens and DAMPs to prime anti‐tumor immunity. This dual cell death cascade remodels the immunosuppressive TME via boosted DC maturation and CD8^+^ T cell infiltration, synergizing with anti‐PD‐L1 therapy to reverse checkpoint resistance. Further tumor rechallenge and memory T cell profiling experiments confirmed that the combination of radiotherapy and this nanovaccine elicits robust, long‐lived systemic anti‐tumor immune memory dominated by expanded central and effector memory CD8^+^ T cells, which provides memory‐like protection against tumor recurrence and secondary tumor engraftment. Collectively, this work establishes a clinically translatable radiotherapy‐initiated in situ nanovaccination strategy based on ferroptosis‐ICD crosstalk for refractory PAAD and other immunosuppressive tumors.

## 5 Materials and methods

### 5.1 Materials and Instrumentation

DPPC (LP‐R4‐057, Ruixibio, China), DOTAP (LP‐R4‐117, Ruixibio, China), cholesterol (R‐H‐100001, Ruixibio, China), DSPE‐PEOz (R‐PL2056‐Ig, Ruixibio, China), DSPE‐PEG_2_K‐cRGD (R‐9995, Ruixibio, China), and Cy5.5 (R‐CY2012, Ruixibio, China). DMEM or RPMI‐1640 medium (Gibco, Thermo Fisher, USA) containing 10% fetal bovine serum (Gibco, Thermo Fisher, USA) and 1% G5 supplement (Thermo Fisher, USA) was used for cell culture. TRIzol Reagent (Invitrogen, Carlsbad, USA) was applied for total RNA extraction. CCK‐8 reagent (MedChemExpress, HY‐K0301) was utilized for cell viability assay. Crystal violet (G1014; Servicebio) was used for cell staining. EdU cell proliferation assay kit (C0071, Beyotime, China), 1× Hoechst 33342 reagent (C1011, Beyotime, China), and Live/Dead Cell Staining Kit (C2015, Beyotime, China) were adopted for cellular phenotype detection. PrimeScript RT Reagent Kit (Takara, Otsu, Japan) and Super SYBR Green qPCR Master Mix Kit (Esscience, China) were used for reverse transcription and quantitative PCR, respectively. RIPA lysis buffer (PC102, Epizyme) supplemented with PMSF (GRF101, Epizyme) and phosphatase inhibitors (GRF102, Epizyme) was prepared for protein extraction, and polyvinylidene fluoride (PVDF) membranes (WJ002, Epizyme) were used for western blot. Cell Ferrous Iron (Fe^2^^+^) Fluorometric Assay Kit (MA0647, Meilunbio, China), BODIPY 581/591 C11 (D3861, Thermo Fisher, USA), and Reactive Oxygen Species Assay Kit (S0033, Beyotime, China) were employed to evaluate ferroptosis‐related parameters. Gel‐Red (D0140, Beyotime) and DAPI (D1006, Beyotime, China) were used for nucleic acid staining. InVivoMAb anti‐mouse PD‐L1 (B7‐H1) antibody (clone 10F.9G2, BioXCell, Cat# BE0101) and gemcitabine (MedChemExpress, HY‐17026) were used for in vivo treatment.

Synergy H1 (SH1M) microplate reader (Agilent‐BioTek, USA), Nanodrop spectrophotometer (Thermo Fisher, USA), ChemiDoc XRS+ imaging system (Bio‐Rad, USA), and SH800S cell sorter (Sony Biotechnology Company, USA) were used for sample detection and analysis. Dynamic light scattering (DLS) measurements were performed using a Malvern Zetasizer Nano ZS. A transmission electron microscope (TEM; H‐7000, Hitachi, Japan) was used to characterize nanoparticle morphology. A fluorescence microscope (Eclipse Ts2R, Nikon, Japan) was applied for cellular fluorescence imaging. A confocal microscope (TCS SP5, Leica, Germany) was used for high‑resolution cellular fluorescence observation.

### 5.2 Differentially Expressed Genes (DEGs) and Enrichment Analyses

The mRNA sequencing data and clinical information of patients with PAAD were obtained from the TCGA, GEO, and ArrayExpress databases. Differential expression genes (DEGs) were screened via the limma R package with the cutoff thresholds of adjusted *p*‐value < 0.05 and |log fold change (logFC)| > 1. Gene Ontology (GO) and Kyoto Encyclopedia of Genes and Genomes (KEGG) enrichment assessments were performed to characterize DEGs and probe their underlying functional modules as well as regulatory signaling cascades (adjusted *p* < 0.05).

Gene Set Variation Analysis (GSVA) is a specialized gene set enrichment method designed to quantify scores for specific gene sets and perform differential analysis at the gene set (pathway) level [40]. Ferroptosis‐related gene set data were retrieved from the Molecular Signatures Database (MSigDB; available at: http://software.broad‐institute.org/gsea/msigdb). Subsequently, the “GSVA” R package (version 4.16) was utilized to calculate the score of each sample across individual ferroptosis‐related pathways, thereby evaluating the corresponding biological functions.

### 5.3 Analysis of Single‐Cell RNA Sequencing Data

This study integrated and analyzed four pancreatic cancer scRNA‐seq public datasets (CRA001160, GSE155698, GSE197177, and GSE205013). The Seurat R package was implemented for single‐cell data processing. Potential doublets were excluded by removing cells with aberrantly high gene counts (> 7000). High‐quality cells (500–50 000 UMI/cell, 500–7000 genes/cell, < 20% mitochondrial genes, and < 5% hemoglobin genes) were retained, yielding a refined dataset of 196 829 tumor cells for downstream analysis. Cell‐level normalization was performed using the NormalizeData function. The top 3000 highly variable genes were selected using the Find Variable Features function. Batch effects across different cohorts were corrected with the Harmony R package. The shared nearest neighbor (SNN) graph was constructed using the Find Neighbors function (top 30 PCs), and cell clusters were identified using the Louvain algorithm (resolution = 0.5). Clusters were annotated using known markers: T cells (CD3D, CD3E, CD2, CD3G); NK cells (KLRB1, NKG7, CD161); B cells (MS4A1, CD79A, CD79B, CD19); Plasma cells (CD38, CD27, TNFRSF17); Monocytes (S100A8, FCN1, CD14); Macrophages (CD68, AIF1); Mast cells (TPSAB1, HPGDS, MS4A2); Fibroblasts (COL1A1, ACTA2, DCN); Endothelial cells (CD36, FLT1, VWF); and Epithelial cells (EPCAM, CDH1, KRT19). Malignant tumor cells were specifically distinguished from normal epithelium by the high expression of KRAS and MKI67. To evaluate antigen presentation capacity, MHC‐I (HLA‐A/B/C, B2M, TAP1/2, PSMB8/9, CALR) and MHC‐II (HLA‐DPA1/DQB1/DRA/DRB1, CD80/86/40, CIITA) signature scores were calculated using the AddModuleScore function. Differences between FSTL3‐high and FSTL3‐low tumor cells were assessed using the Wilcoxon rank‐sum test.

### 5.4 Random Forest Analysis

The random forest classifier was implemented using the RandomForestSRC R package (version 3.3.3). The model was trained with ntree = 1000 trees, and the mtry parameter (number of variables considered at each split) was set to sqrt(ncol(data)). Model performance was evaluated using 5‐fold cross‐validation, and the out‐of‐bag (OOB) error rate was monitored to confirm convergence. Variable importance was quantified using the Gini impurity criterion, which measures the total reduction in node impurity contributed by each feature across all trees. Raw importance scores are presented in Figure 1C.

### 5.5 RNA Isolation and Sequencing Analysis

Total RNA was extracted from the tissue using TRIzol Reagent (Invitrogen, Carlsbad, USA) according to the manufacturer's instructions. RNA purification, reverse transcription, library construction, and sequencing were performed at Hangzhou Cosmos Wisdom Biotechnology Co., Ltd. (Hangzhou, China) according to the manufacturer's instructions (Illumina, San Diego, CA). The DEGs were derived from various cell samples (PANC‐1R and PANC‐1). DEGs with |log2FC| ≧ 1 and FDR ≤ 0.05 (DESeq2) were considered to be significantly differentially expressed genes.

### 5.6 Cell Culture

Human pancreatic adenocarcinoma (PAAD) cell lines PANC‐1, SUIT‐2, as well as the murine PAAD cell line PANC02, were obtained from the American Type Culture Collection (ATCC, Manassas, VA, USA). Prior to experiments, all cell lines employed in this work were subjected to short‐tandem‐repeat (STR) profiling for identity authentication. Routine mycoplasma testing was performed to verify that no mycoplasma contamination existed in all cultured cell lines. Cells were maintained in DMEM medium (Gibco, Thermo Fisher, USA) supplemented with 10% fetal bovine serum (Gibco, Thermo Fisher, USA) and 1% G5 supplement (Thermo Fisher, USA) within a humidified incubator set at 37°C with 5% CO_2_.

### 5.7 Cell Proliferation/Toxicity

Human PANC‐1 and SUIT‐2 cells were trypsin‑digested and resuspended, followed by seeding into 96‐well plates at 6000 cells per well in a total volume of 100 µL cell suspension. Once cells attained full adherence, diverse experimental interventions were applied, and cells were further incubated for 24 h. Subsequently, culture medium was substituted with complete DMEM supplemented with 10% CCK‐8 reagent, and cells were incubated for 1–2 h. Optical density values were recorded at 450 nm using a Synergy H1 (SH1M) microplate reader (Agilent‐BioTek, USA).

### 5.8 Colony Formation Assay

Human pancreatic cancer cells (PANC‐1 and SUIT‐2) in the logarithmic growth phase (200, 400, 800, 1600/well) were seeded into a 6‐well plate and treated with different doses of irradiation (0, 2, 4, and 6 Gy) in triplicate. After irradiation, cells were cultured for 7–12 days to form colonies and then fixed and stained with crystal violet (G1014; Servicebio). The colonies (with > 50 cells) were counted for the survival fraction calculation, and the survival curves were fitted with the formula SF = 1‐(1‐exp(−k*D))ˆN based on the single‐hit multi‐targeted model. The sensitization enhancement ratio (SER) was calculated based on the radiation dose required to reduce cell survival to 10% (D_10_), derived from the fitted survival curves. SER was defined as the ratio of the D_10_ of the control group to the D_10_ of the treatment group.

### 5.9 Establishment of Radioresistant Cell Line

Radioresistant cell lines were established by exposing parental cells to fractionated x‐ray irradiation (6 Gy per fraction, once weekly, for a total of 10 fractions, resulting in a cumulative dose of 60 Gy). The radioresistant phenotype was validated by colony formation assays across a range of radiation doses (0, 2, 4, 6 Gy), with each experiment performed in three independent replicates.

### 5.10 EdU Experiment

The proliferation of cells was detected using the EdU cell proliferation assay according to the manufacturer's instructions (C0071, Beyotime, China). Briefly, cells were incubated with EdU for 2 h to label them. After three times washing with PBS, cells were fixed in a 4% paraformaldehyde solution for 15 min, permeabilized with 0.3% Triton X‐100 for another 15 min, and then incubated with the click‐reaction reagent for 30 min at room temperature in a dark environment. Then, 1× Hoechst33342 reagent (C1011, Beyotime, China) was used to counterstain the nucleus. The result of staining was observed with a fluorescence microscope (Eclipse Ts2R, Nikon, Japan).

### 5.11 Live/Dead Staining Assay

Cells were seeded in 24‐well culture plates and incubated in a cell incubator at 37°C for 24 h. Subsequently, 500 µL of medium containing nanoparticles was added, and cells were incubated for 24 h. Then, cells were irradiated with 6 Gy x‐rays and continued to culture for another 24 h. After PBS washes, cells were stained with 500 µL of staining solution from the Live/Dead Cell Staining Kit (C2015, Beyotime, China) and incubated in the dark at 37°C for 20 min. After 3 washes with PBS, cells were immediately observed under a fluorescence microscope (Eclipse Ts2R, Nikon, Japan).

### 5.12 Real‐Time Quantitative Polymerase Chain Reaction (RT‐qPCR)

Total cellular RNA was isolated with TRIzol Reagent (Invitrogen, Carlsbad, USA), and RNA concentration was quantified by a Nanodrop spectrophotometer (ThermoFisher, USA). Purified RNA was reverse‐transcribed into complementary DNA employing the PrimeScript RT Reagent Kit (Takara, Otsu, Japan). qPCR amplification was carried out with the Super SYBR Green qPCR Master Mix Kit (Esscience, China). The sequences of the specific primers are shown (Table S2).

### 5.13 Western Blot

Total protein was harvested from differently treated PANC‐1 and SUIT‐2 cells with RIPA lysis buffer (PC102, Epizyme) supplemented with PMSF (GRF101, Epizyme) and phosphatase inhibitors (GRF102, Epizyme). Protein samples were resolved by SDS‐PAGE and electro‐transferred onto polyvinylidene fluoride (PVDF) membranes (WJ002, Epizyme). Membranes were blocked with 5% BSA for 1 h, followed by overnight incubation with primary antibodies at 4°C. Afterward, membranes were probed with matching secondary antibodies for 1 h at room temperature. Immunoblot signals were captured with a ChemiDoc XRS+ imaging system (Bio‐Rad, USA). Antibody information is compiled in Table S3.

### 5.14 Intracellular Ferrous Ions (Fe^2+^) Detection

Cell Ferrous Iron (Fe^2+^) Fluorometric Assay Kit (MA0647, Meilunbio, China) was used to detect the content of Fe^2+^ in PANC‐1 and SUIT‐2 cells in different treatment groups according to the product instructions. Briefly, after digestion and collection, the cells were washed with PBS and counted. The cells were then incubated with an Fe^2^^+^ probe at 37°C in a cell‐culture incubator for 30 min, followed by three PBS washes. The fluorescence intensity of the reaction product was measured using a Synergy H1 fluorescence microplate reader (Agilent BioTek, USA) at Ex/Em = 543/580 nm.

### 5.15 Lipid Peroxidation Detection

PANC‐1 and SUIT‐2 cells were harvested and resuspended in serum‐free DMEM medium. BODIPY 581/591 c11 (D3861, Thermofisher, USA) was added to the cell suspension and incubated at 37°C for 30 min. The labeled cells were analyzed by the SH800S cell sorter (Sony Biotechnology Company, USA). The obtained data were processed by using Flow Jo version 10.8 software (BD Biosciences).

### 5.16 ROS Detection

Treated cells from different groups were harvested and washed with PBS. Then, DCFH‐DA from the Reactive Oxygen Species Assay Kit (S0033, Beyotime, China) was loaded for the detection of ROS in cells at a concentration of 10 µm, and incubated in the dark at 37°C for 20 min. After 3 washes with PBS, the fluorescence intensity was measured using a Synergy H1 fluorescence microplate reader (Agilent BioTek, USA) at an excitation wavelength of 488 nm and an emission wavelength of 525 nm.

### 5.17 Flow Cytometry

Tumor samples were digested and processed to single‐cell suspensions, followed by red blood cell lysis. For memory T cell detection, mouse spleens were harvested, mechanically ground, and filtered to prepare splenic single‐cell suspensions before erythrocyte lysis. Following erythrocyte lysis, cell suspensions were stained with surface‐marker antibodies in the dark for 30 min. For intracellular staining, cells were further fixed, permeabilized, and incubated with corresponding intracellular antibodies. The immune‑cell immunophenotyping panels were defined as follows: DCs (CD45^+^CD11c^+^MHC‑II^+^), CD8^+^ T cells (CD45^+^CD3^+^CD8^+^), CD4^+^ T cells (CD45^+^CD3^+^CD4^+^). For splenocyte‑derived CD8^+^ T‑cell memory subsets: naive CD8^+^ T cells (CD44^−^CD62L^+^), central‑memory T cells (T_CM_, CD44^+^CD62L^+^), effector‑memory T cells (T_EM_, CD44^+^CD62L^−^), and effector CD8^+^ T cells (CD44^+^CD62L^−^). Sample acquisition was performed on an SH800S cell sorter (Sony Biotechnology, USA). Flow cytometry datasets were processed and visualized with FlowJo software (version 10.8.1). Full antibody specifications are summarized in (Table S4). The stepwise gating workflow for immune cell immunophenotyping by flow cytometry is presented in Figures S21 and S22.

### 5.18 Synthesis and Characterization of Nanovaccines

The materials used for the synthesis of cationic nanovesicles, including DPPC (LP‐R4‐057, Ruixibio, China), DOTAP (LP‐R4‐117, Ruixibio, China), cholesterol (R‐H‐100001, Ruixibio, China), DSPE‐PEOz (R‐PL2056‐Ig, Ruixibio, China), and DSPE‐PEG2K‐cRGD (R‐9995, Ruixibio, China), were mixed at a specific mass ratio and dissolved in a chloroform‐methanol mixture. The resulting solution was placed in a rotary evaporator at 45°C for 15 min to form a thin film. The obtained liposome suspension underwent repeated extrusion through 100 nm polycarbonate membranes with a liposome extruder. The obtained cationic liposome was used for subsequent loading of siRNA.

Dynamic Light Scattering (DLS) measurements were performed on a Malvern Zetasizer Nano ZS to determine the size, zeta potential, and polydispersity index (PDI) for evaluating the homogeneity and stability of the synthesized cationic nanovesicles. A transmission electron microscope (TEM; H‐7000 Electron Microscope, Hitachi, Japan) was employed to assess the nanomaterials’ size and morphology.

### 5.19 Determination of Encapsulation Efficiency by the Ultrafiltration Method

The encapsulation efficiency (EE) of nanoliposomes was determined using the centrifugal ultrafiltration method. Briefly, an appropriate volume of liposome suspension was loaded into a pre‐rinsed ultrafiltration tube with a 10 kDa molecular weight cutoff (MWCO) membrane. The sample was centrifuged at 6000 rpm for 15 min to separate free drug from liposome‐encapsulated drug. Free small‐molecule drug penetrated through the ultrafiltration membrane, while intact liposome nanoparticles were retained in the upper chamber.

The obtained filtrate was collected and analyzed via high‐performance liquid chromatography (HPLC) to quantify the concentration of free drug (C_free_). In parallel, an equal volume of fresh liposome suspension was fully demulsified and disrupted using methanol with ultrasonic treatment to completely release the encapsulated drug. The total drug concentration (C_total_) was determined by HPLC under identical detection conditions. The encapsulation efficiency was calculated according to the following formula: EE% = (C_total_‐C_free_)/ C_total_ x 100%.

### 5.20 Gel Retardation Assay

A 2% agarose gel was made by dissolving 2 g agarose powder in 100 mL of 1× TBE buffer under gentle agitation. The mixture was microwaved to achieve full agarose dissolution. Upon cooling to approximately 60°C, 10 µL of Gel‐Red (D0140, Beyotime, China) was supplemented and mixed gently, followed by pouring the solution into a gel‐casting mold. Samples from distinct experimental groups were loaded into separate gel wells. Electrophoresis was run at 100 V for 40 min. Gel images were acquired with the ChemiDoc XRS+ imaging system (Bio‑Rad, USA) to assess sample migration profiles.

### 5.21 Cellular Uptake Assay

Cells were seeded onto a confocal petri dish at a density of 1.5 × 10^5^ cells per well and incubated with Cy5.5‐siNC/Lip‐cRGD for 6, 12, and 24 h. After incubation, cells were fixed with 4% paraformaldehyde for 15 min, and nuclei were stained with DAPI (D1006, Beyotime, China) for 5 min. After 3 washes with PBS, cellular uptake was visualized using a Leica TCS SP5 confocal microscope (Leica, Germany).

### 5.22 In Vivo Uptake Study

Subcutaneous PANC02 tumor‐bearing C57BL/6 mice (tumors located at the right abdominal or axillary site) were assigned randomly to two experimental cohorts (n = 3). Free Cy5.5 (R‐CY2012, Ruixibio, China) and Cy5.5‐Lip‐cRGD were delivered via tail‐vein intravenous injection in corresponding groups. Serial in vivo optical imaging was acquired at 2, 6, 12, and 24 h post‐injection on the IVIS Spectrum platform. Upon 24 h post‐treatment, mice underwent euthanasia; heart, liver, spleen, lung, kidney and tumor tissues were excised to examine the in vivo biodistribution profile. Living Image software (v4.4, PerkinElmer, USA) was utilized to process and analyze imaging datasets.

### 5.23 Cell and Tumor Irradiation

PAAD cells were irradiated with 6‐MV x‐rays using a linear accelerator (Elekta, Sweden) at 200 cGy/min. Irradiation was performed at the Experimental Irradiation Core of the Naval Medical University, Shanghai, China. The distance between the 6‐well plate and the radiation source was 100 cm, and the radiation field was 30 cm×30 cm. For in vivo tumor irradiation, mice with PAAD syngeneic tumors were anesthetized with CO_2_ and placed in a customized lead mould to receive localized x‐ray irradiation at a single dose of 6 Gy, which was administrated 24 h after the initial intravenous injection of liposomal nanoparticles.

### 5.24 BMDC Extraction, Induction, and Co‐Culture With Treated PANC02 Cells

Bone marrow was isolated from femurs and tibias of 8‐week‐old C57BL/6 mice. Marrow tissues were filtered and triturated to prepare single‐cell suspensions. Upon centrifugation, the supernatant was aspirated, and the cell pellet was treated with red blood cell lysis buffer for 3 min. PBS was supplemented to stop lysis, and cells were pelleted and washed once more before plating. Cells were differentiated into BMDCs over 7 days in RPMI‐1640 medium supplemented with 20 ng mL^−^^1^ GM‐CSF (RP01206, ABclonal, China) and 10 ng mL^−^^1^ IL‐4 (RP01161, ABclonal, China). Differentiated BMDCs were co‐incubated with PANC02 cells receiving distinct treatments in Transwell inserts at a 2:1 cell ratio. Forty‐eight hours later, BMDCs were harvested, and the proportion of mature DCs was quantified via flow cytometry using the SH800S cell sorter (Sony Biotechnology Company, USA).

### 5.25 Experiments In Vivo

Six‐week‐old female C57BL/6 mice were obtained from Charles River Lab. Animal Co. Ltd. (Beijing, China). Animals were kept in a controlled environment with a 12 h light/dark photoperiod, ambient temperature maintained at 22°C ± 2°C, and food and water provided ad libitum. After one‐week acclimation period, mice were randomly assigned for subcutaneous tumor inoculation. A total of 200 µL of PANC02 cell suspension at a concentration of 2 × 10^6^ cells mL^−1^ was injected subcutaneously into the right abdomen or armpit of each mouse. When the tumor volume reached ≈100 mm^3^, mice were randomly divided into 5 groups according to the treatment plans (G1: Lip‐cRGD, IR+G1, IR+G2: IR + siFSTL3/Lip‐cRGD, IR+G3: IR + siNC/Lip_GEM_‐cRGD, IR+G4: IR + siFSTL3/Lip_GEM_‐cRGD) (n = 5). On day 13, after 24 h of nanovaccine administration through the caudal vein, an x‐ray at a dose of 6 Gy was performed on the tumor. The nanovaccines were administered 4 times during the whole experimental period, weekly. In the experiment of combination therapy with aPD‐L1, mice bearing subcutaneous PANC02 cell tumor were randomly divided into five groups (n = 5): G1 + vector, G1 + aPD‐L1, G1 + aPD‐L1 + IR, G4 + aPD‐L1, G4 + IR + aPD‐L1. aPD‐L1 (InVivoMAb anti‑mouse PD‐L1 (B7‐H1), clone 10F.9G2, BioXCell, Cat# BE0101) was administered intraperitoneally at a dose of 10 mg/kg weekly for a total of 3 doses. The nanoparticles were administered 3 times during the whole experimental period, weekly. On day 11, 24 h following the initial intravenous (caudal vein) injection of liposomal nanoparticles, the tumor was subjected to 6 Gy x‐ray irradiation.

To evaluate memory‐like adaptive antitumor immunity induced by irradiation combined with G4 nanovaccine, we constructed a pancreatic tumor rechallenge model in immunocompetent mice. Briefly, 2 × 10^6^ viable PANC02 cells suspended in PBS were subcutaneously injected into the left lateral thigh of each mouse on day 0 to establish primary tumors (n = 5). Mice received local radiotherapy and sequential intravenous G4 nanovaccine administration at indicated timepoints. All primary PANC02 tumors were surgically resected on day 29. For tumor rechallenge on day 40, 5 × 10^5^ PANC02 cells were subcutaneously inoculated into the contralateral (right) lateral thigh. Mice were monitored daily for survival until day 60 post‐rechallenge.

Mouse body weight was recorded every three days, and tumor dimensions (length [L] and width [W]) were measured using a vernier caliper every three days. Tumor volume was calculated using the formula V = (L × W^2^) / 2. At the end of the experiment, mice were euthanized using CO_2_, and tissues, including the heart, liver, spleen, lungs, kidneys, and tumors, were harvested for subsequent analysis. A portion of the excised tumor tissue was fixed in 4% paraformaldehyde for histological analysis; the remaining tissue was stored at −80°C for subsequent experimental studies. All animal studies were approved by the Ethics Committee of Naval Medical University, Shanghai, China (Approved number: 82272742). All animal experimental procedures were carried out in strict accordance with the ARRIVE guidelines and the institutional guidelines for the care and use of laboratory animals.

### 5.26 Immunohistochemistry (IHC) and Immunofluorescence (IF) Staining Assays

Tumor tissues from different treatment groups were subjected to dehydration, paraffin embedding, and serial sectioning to prepare paraffin‐embedded sections. After dewaxing, the sections were processed for antigen retrieval and non‐specific binding blocking. Subsequently, the sections were incubated with the corresponding primary antibodies overnight at 4°C. On the following day, the sections were rinsed thoroughly with phosphate‐buffered saline (PBS), followed by incubation with fluorophore‐conjugated secondary antibodies for 1 h at room temperature in the dark. For signal visualization, the sections were incubated with 3,3'‐diaminobenzidine (DAB) or TAB chromogen. Nuclear counterstaining was then performed using DAPI. Finally, the sections were mounted and imaged under a fluorescence microscope. All patient‐derived tumor tissues used in this study were obtained with ethical approval from the Ethics Committee of Changhai Hospital affiliated to Naval Medical University, Shanghai, China (Ethics No. CHEC2023‐037). Written informed consent was acquired from all participating patients.

### 5.27 Statistical Analysis

All assays were performed with three independent biological replicates. Statistical computations were implemented with GraphPad Prism v10.4 and R software. Quantitative datasets were reported as mean ± SD. Multiple‐group comparisons were assessed via one‑way ANOVA, and two‑group comparisons were conducted using the unpaired Student's *t*‐test. *P* < 0.05 was considered statistically significant.

## Author Contributions

Conceptualization: H.Z., B.T. and Z.M. Methodology: C.H., X.L. and C.W. Investigation: X.L., X.Z. and D.C. Visualization: C.H. and Y.H. Supervision: X.L., X.Z., Y.G., L.C. and W.C. Writing – original draft: C.H. and Y.H. Writing – review & editing: H.Z., B.T. and Z.M. The authors declare that the DeepSeek large‐language‐model AI tool was utilized solely for manuscript language polishing and grammatical error checking. No AI was involved in generating experimental data, designing figures, analyzing experimental results or establishing scientific conclusions. All experimental work, data interpretation and scientific reasoning were completed exclusively by the authors.

## Funding

This study was funded by National Natural Science Foundation of China (82272742, 82173743, 82473233 and 82303680)and Shanghai Health Commission Leading Talent Program(2022LJ019).

## Conflicts of Interest

The authors declare no conflicts of interest.

## Supporting information




**Supporting File**: advs77651‐sup‐0001‐SuppMat.docx.

## Data Availability

The raw RNA‐seq sequencing data generated in this study have been deposited in the NCBI Sequence Read Archive (SRA) under the accession number PRJNA1514647 and PRJNA1514983. The data that support the findings of this study are available from the corresponding author upon reasonable request.
