# Supplementary material for: Multifunctional Nanovaccine‐Radiotherapy Synergy Drives Anti‐Tumor Immunity via Multi‐Cascade Cell Death for Pancreatic Cancer Therapy
Source: Adv Sci (Weinh). 2026 Sep 10:e77651. Online ahead of print. doi: 10.1002/advs.77651 (PMC13562777; doi:10.1002/advs.77651)
Supplement: Supplementary file 1 [file ADVS-9999-e77651-s001.docx]

**Supplementary Materials**

**Multifunctional nanovaccine-radiotherapy synergy drives anti-tumor immunity via multi-cascade cell death for pancreatic cancer therapy**

Chengyi Huang^1^†, Yushu Han^1^†, Xiang Liu^4^†, Chuanhao Wang^2^, Xiaofei Zhu^1^, Di Chen^1^, Yangyang Gong^1^, Liang Chen^1^, Wenjuan Chen^1^, Zhenyuan Miao^2^*, Bufu Tang^3^*, Huojun Zhang^1^*

**^1^** Department of Radiation Oncology, Changhai Hospital affiliated to Naval Medical University, Shanghai 200433, China. **^2^** School of Pharmacy, Naval Medical University, Shanghai 200433, China.

**^3^** Department of Interventional Radiology, Zhongshan Hospital, Shanghai Institute of Medical Imaging, Shanghai Institution of Medical Imaging, Shanghai, National Clinical Research Center of Interventional Medicine, Fudan University, Shanghai 200032, China

**^4^** Department of Oncology, Zhujiang Hospital, Southern Medical University, Guangzhou, Guangdong, 510280, China.

†**:** These authors contributed equally to this work.

Corresponding authors:

Prof. Huojun Zhang, E-mail: [chyyzhj@163.com](mailto:chyyzhj@163.com)

Prof. Bufu Tang, E-mail: [tangbufu@zju.edu.cn](mailto:tangbufu@zju.edu.cn)

Prof. Zhenyuan Miao, E-mail: miaozhenyuan@hotmail.com

**SUPPLEMENTARY TABLE**

Table S1. The target sequence of siRNA

| siRNA | Species | Sense sequence (5’ to 3’) | Antisense sequence (5’ to 3’) |
| --- | --- | --- | --- |
| siFSTL3-1 | Human | GAAGAGAACUUCGUGUGAGTT | CUCACACGAAGUUCUCUUCTT |
| siFSTL3-2 | Human | CACAGCAGAGUCUAAUUUATT | UAAAUUAGACUCUGCUGUGTT |
| siFSTL3-1 | Mus | CAGCAGAGGAGGAAGAGAATT | UUCUCUUCCUCCUCUGCUGTT |
| siFSTL3-2 | Mus | AGUCGUGCCUUGUGGAUCATT | UGAUCCACAAGGCACGACUTT |
| NC | Human/Mus | UUCUCCGAACGUGUCACGUTT | ACGUGACACGUUCGGAGAATT |

Table S2. Primers used in the research

| Primers | Species | Forward | Reverse |
| --- | --- | --- | --- |
| FSTL3 | Human | ACTGCGTGGTGTGTCGAG | TGGCACGAGGAGATGTAGGT |
| GAPDH | Human | GGAGCGAGATCCCTCCAAAAT | GGCTGTTGTCATACTTCTCATGG |
| FSTL3 | Mus | CACCTACCGGGACGAATGC | CCTGAGCGCAAGACTTTTGAC |
| GAPDH | Mus | AGGTCGGTGTGAACGGATTG | TGTAGACCATGTAGTTGAGGTCA |

Table S3. Antibodies used in the research

| Names | Company | Cat number | Dilution | Species |
| --- | --- | --- | --- | --- |
| FSTL3 | Abcepta | AP12300b | WB:1:2000  IHC: 1:300  IF 1:200 | Rabbit |
| GPX4 | Proteintech | 67763-1-Ig | WB: 1:2000  IF 1:200 | Mouse |
| HMGB1 | Proteintech | 10829-1-AP | WB 1:10000  IF 1:200 | Rabbit |
| ASCL4 | Proteintech | 22401-1-AP | WB:1:10000 | Rabbit |
| GAPDH | Abcam | ab181602 | WB:1:10000 | Rabbit |
| Ki-67 | Proteintech | 28074-1-AP | IHC:1:1000 | Rabbit |
| Calreticulin | Cell Signaling Technology | 12238-S | WB:1:1000  IF:1:500 | Rabbit |
| 4-Hydroxynonenal | Proteintech | 68538-1-Ig | IF 1:1000 | Mouse |
| β-Actin | Abcam | ab8227 | WB 1：10000 | Rabbit |
| STAT1 | Affinity | AF6300 | WB 1:1000 | Rabbit |
| STAT1 | Proteintech | 66545-1-Ig | IF 1:500 | Mouse |
| P-STAT1 | Affinity | AF3300 | WB 1:1000  IF 1:200 | Rabbit |
| IRF1 | Proteintech | 11335-1-AP | WB 1:1000 | Rabbit |
| CD8a | Cell Signaling Technology | 98941 | IF 1:1000 | Rabbit |
| GZMB | Abcam | ab255598 | IF 1:200 | Rabbit |
| PD-L1 | Proteintech | 66248-1-Ig | IHC 1:2000 | Mouse |
| Goat anti Rabbit  IgG-HRP | ABclonal  Technology | AS014 | WB 1:10000 | Goat |
| Goat anti Mouse  IgG-HRP | ABclonal  Technology | AS003 | WB 1:10000 | Goat |

Table S4. FACS antibodies

| Name | Fluorophore | Clone | Company | Cat number |
| --- | --- | --- | --- | --- |
| CD45 | AF700 | 30-F11 | Invitrogen | 56-0451-82 |
| CD3 | PE | 17A2 | BioLegend | 100206 |
| CD3 | APC/Cyanine7 | 17A2 | BioLegend | 100222 |
| CD4 | FITC | GK1.5 | BioLegend | 100406 |
| CD8 | PerCP/Cyanine5.5 | 53-6.7 | BioLegend | 100734 |
| CD11c | FITC | N418 | BioLegend | 117305 |
| I-A/I-E | APC | M5/114.15.2 | BioLegend | 107628 |
| CD80 | APC | 16-10A1 | BioLegend | 104714 |
| CD86 | PE | A17199A | BioLegend | 159204 |
| CD62L | APC | W18021D | BioLegend | 161218 |
| CD44 | PE | IM7 | Biolegend | 103008 |

Table S5. Quantification of unencapsulated gemcitabine (GEM), encapsulated GEM and encapsulation efficiency of GEM-loaded nanoparticles across three independent preparation batches

| Independent repeated experiments | Free unencapsulated GEM (μg) | Total GEM after demulsification (μg) | Encapsulated GEM (μg) | Encapsulated efficiency (EE, %) |
| --- | --- | --- | --- | --- |
| 1 | 16.0461 | 74.6101 | 58.5640 | 78.49 |
| 2 | 17.4686 | 93.6864 | 76.2178 | 81.35 |
| 3 | 19.4114 | 88.2340 | 68.8226 | 78.00 |

Table S6. Quantification of free siRNA, encapsulated siRNA and encapsulation efficiency of siRNA-loaded nanoparticles across three independent replicate experiments.

| Independent repeated experiments | Free unencapsulated siRNA (μg) | Initial total siRNA input (μg) | Encapsulated siRNA (μg) | Encapsulated efficiency (EE, %) |
| --- | --- | --- | --- | --- |
| 1 | 0.0093 | 0.1000 | 0.0907 | 90.71 |
| 2 | 0.0059 | 0.1000 | 0.0941 | 94.06 |
| 3 | 0.0081 | 0.1000 | 0.0919 | 91.88 |

**SUPPLEMENTARY FIGURES**

**Figure S1. Validation of radiation-resistant cell lines.** (A) Clonogenic assay and dose-response analysis of survival fraction (B) in PANC-1 and PANC-1R cells following ionizing radiation (IR).

**Figure S2. FSTL3 expression is significantly elevated in pancreatic adenocarcinoma (PAAD) tissues and correlates with poor prognosis in patients with pancreatic cancer.** (A-B) Kaplan-Meier survival analysis of overall survival (OS) stratifying patients with PAAD into high and low FSTL3 expression groups in the E_MTAB_6134 and GSE71729 cohort. (C) Kaplan-Meier survival analysis of disease-free survival (DFS) stratifying patients with PAAD into high and low FSTL3 expression groups in the TCGA PAAD cohort. (D) Kaplan-Meier survival analysis of progressive-free survival (PFS) stratifying patients with PAAD into high and low FSTL3 expression groups in the TCGA PAAD cohort. (E) Kaplan-Meier survival analysis of relapse-free survival (RFS) stratifying patients with PAAD into high and low FSTL3 expression groups in the E_MTAB_6134 cohort. (F) Quantitative analysis of FSTL3 expression levels in adjacent non-tumor and tumor tissues from PAAD patients. **P* < 0.05.

**Figure S3. Knockdown of FSTL3 in PANC-1 and SUIT-2 PAAD cells using siRNA.** (A) RT-qPCR validation of the knockdown efficiency of two siRNA sequences in PANC-1 and SUIT-2 cells. **P* < 0.05, ***P* < 0.01, ****P* < 0.001. (B) Western blot analysis of FSTL3 protein expression in both cell lines after treatment with siNC, siFSTL3#1, and siFSTL3#2.

**Figure S4. Silencing FSTL3 enhances radiation-induced cytotoxicity in pancreatic cancer cells, which is rescued by FSTL3 overexpression.** (A-B) Cell viability of PANC-1 (A) and SUIT-2 (B) cells receiving siNC, siFSTL3, siNC+IR, siFSTL3+IR, or siFSTL3+p-FSTL3-Res+IR treatment. **P* < 0.05, ***P* < 0.01, ****P* < 0.001.

**Figure S5. FSTL3 overexpression rescues gemcitabine sensitivity induced by FSTL3 silencing in pancreatic cancer cells.** (A-B) Gemcitabine dose-response viability curves of the siFSTL3+p-FSTL3-Res rescue group in PANC-1 (A) and SUIT-2 (B) cells. (C-D) Statistical comparison of gemcitabine IC50 among siNC, siFSTL3, and siFSTL3+p-FSTL3-Res groups in PANC-1 (C) and SUIT-2 (D) cells. Data are presented as mean ± SD; **P* < 0.05, ***P* < 0.01, ****P* < 0.001.

**Figure S6. Heatmap of top two representative marker genes for each cell cluster identified by single-cell RNA-seq in pancreatic cancer tissues.** Rows correspond to cell-type-specific marker genes, and columns represent distinct cell populations, including T cells, NK cells, B cells, plasma cells, monocytes, macrophages, mast cells, endothelial cells, fibroblasts, normal epithelial cells and tumor epithelial cells. The color gradient from blue to red indicates the normalized relative gene expression level ranging from −3 to 3, where red denotes high expression and blue denotes low expression.

**Figure S7. Schematic workflow of the in vitro co-culture system using PANC-02 pancreatic cancer cells and bone marrow-derived dendritic cells (BMDCs).** Bone marrow cells isolated from mouse femurs and tibias were induced to differentiate into BMDCs with GM-CSF and IL-4 for 7 days. PANC-02 cells were divided into four treatment groups: siNC, siFSTL3, siNC combined with irradiation (IR), and siFSTL3 combined with IR. After corresponding interventions, treated tumor cells were co-cultured with mature BMDCs for subsequent immune phenotype analysis.

**Figure S8. Validation of FSTL3 knockdown efficiency in PANC-02 cells.** (A) qPCR analysis of relative FSTL3 mRNA expression in PANC-02 cells transfected with negative control siRNA (siNC) or two independent FSTL3-targeting siRNAs (siFSTL3-1, siFSTL3-2). Data are presented as mean ± SD, ****P* < 0.001. (B) Western blot detection of FSTL3 protein levels in PANC-02 cells after siRNA transfection; β-Actin serves as the loading control.

**Figure S9. Particle Size of Different Nanoparticles.** (A) Lip. (B) Lip-cRGD. (C) siNC/Lip-cRGD. (D) Lip_GEM_-cRGD.

**Figure S10. Schematic diagram of the in vitro co-culture system of PANC-02 pancreatic tumor cells and bone marrow-derived dendritic cells (BMDCs) , and flow cytometry analysis of BMDC maturation.** (A) Schematic illustration of the coculture system for PAAD mice PANC-02 cells, and BMDCs. (B) Representative FCM plots showing changes in the proportion of CD80+CD86+ DCs under the indicated treatments.

**Figure S11. Liposome-encapsulated siFSTL3 efficiently knocks down FSTL3 protein expression in PANC-02 cells.** Western blot analysis of FSTL3 protein levels in PANC-02 cells treated with liposome-packaged negative-control siRNA (siNC) or liposome‑encapsulated siFSTL3. β-Actin served as the loading control.

**Figure S12. Differentially expressed genes and GO Analysis in cells treated with IR with or without siFSTL3/Lip_GEM_-cRGD.** (A) Volcano plot illustrating differentially expressed genes in PANC-1 cells after IR treatment with or without siFSTL3/Lip_GEM_-cRGD intervention. (B) GO analysis was performed in the aspect of cellular component (CC). (C) GO analysis was performed in the aspect of molecular function (MF).

**Figure S13. Scatter plot of Spearman correlation analysis for IRF1 and STAT1.** Gene expression values were shown as log₂-transformed TPM. Spearman correlation coefficient R = 0.59, P = 6 × 10⁻¹⁸.

**Figure S14. FSTL3 overexpression reverses G4-induced STAT1 phosphorylation under radiation in PANC-1 and SUIT-2 cells.** Western blot analysis of p-STAT1 and total STAT1 in irradiated PANC-1 (A) and SUIT-2 (B) cells treated with IR+G1, IR+G4, or IR+G4+p-FSTL3-Res. G4 upregulated radiation-triggered p-STAT1, whereas FSTL3 overexpression abrogated this elevation. β-Actin served as loading control.

**Figure S15. Western blot quantification of p-STAT1, IRF1 and ACSL4 in PANC-1 (A) and SUIT-2 (B) cells after IR+G1 or IR+G4 treatment.** Mean ± SD, n=3, **P* < 0.05.

**Figure S16. Flow cytometric detection of lipid peroxidation level (C11-BODIPY fluorescence intensity) in PANC-1 and SUIT-2 pancreatic cancer cells with different treatments.**

(A, B) Representative C11-BODIPY flow histograms of PANC-1 (A) and SUIT-2 (B) cells treated with Blank, G1, G1+IRF1-IN-1, IR+G4, and IR+G4+IRF1-IN-1. (C, D) Representative C11-BODIPY flow histograms of PANC-1 (C) and SUIT-2 (D) cells treated with Blank, G1, G1+LIBX-A401, IR+G4, and IR+G4+LIBX-A401. (E, F) Quantified relative mean fluorescence intensity (MFI) of C11-BODIPY corresponding to panels A and B for PANC-1 (E) and SUIT-2 (F). (G, H) Quantified relative MFI of C11-BODIPY corresponding to panels C and D for PANC-1 (G) and SUIT-2 (H). ***P* < 0.01, ****P* < 0.001. NS, not significant (*P* > 0.05).

**Figure S17. Hematoxylin and eosin (H&E) histological staining of heart, liver, spleen, lung, kidney and tumor tissues in each treatment group.** Scale bar 100 μm.

**Figure S18. In vivo toxicity assessment of various treatment groups.** (A-E) Measurement of ALT, AST, ALB, CK, and BUN levels in mice after different treatments. NS, not significant (*P* > 0.05).

**Figure S19. Quantitative analysis of PD-L1 expression in tumors from the G1 and IR+G4 groups.** **P* < 0.05.

**Figure S20. In vivo toxicity assessment of various treatment groups.** (A-D) Measurement of ALT, AST, ALB, and BUN levels in mice after different treatments. NS, not significant (*P* > 0.05).

**Figure S21. Gating strategy for immune cell subsets identification by flow cytometry.** Total intact cells were gated on FSC-A/SSC-A, followed by singlet filtering with FSC-A/FSC-H. Live cells were enriched by Live/Dead negative selection. CD45⁺ immune cells were identified from viable single cells, then split into CD3⁺ T cells and CD11c⁺MHC-II⁺ DCs. CD8⁺ cytotoxic T cells were gated within CD3⁺ T lymphocytes. Numbers in gates denote percentages relative to the parent gate.

**Figure S22. Flow cytometry gating scheme for characterization of CD4⁺ and CD8⁺ T cell memory phenotypes.** Cells were sequentially gated on total events, singlets, live CD45⁺ leukocytes, and CD3⁺ T cells. CD3⁺ T cells were further separated into CD4⁺ and CD8⁺ populations, which were subsequently stratified into naïve (CD44⁻CD62L⁺), central memory (CM; CD44⁺CD62L⁺), and effector (CD44⁺CD62L⁻) T cell subsets based on CD44 and CD62L expression. Values denote the percentage of cells within the preceding parent gate.
